# Design of Vitrimers with Simultaneous Degradable and Dynamic Crosslinkers: Mechanical and Thermal Behavior Based on Transesterification Reactions Between *β*-Amino Esters and Hydroxylated Acrylate/Methacrylate Monomers

**DOI:** 10.3390/polym17182448

**Published:** 2025-09-10

**Authors:** Naroa Ayensa, Felipe Reviriego, Helmut Reinecke, Alberto Gallardo, Carlos Elvira, Juan Rodríguez-Hernández

**Affiliations:** Instituto de Ciencia y Tecnología de Polímeros (ICTP), CSIC, C/Juan de la Cierva 3, 28006 Madrid, Spain; naroa@ictp.csic.es (N.A.); gallardo@ictp.csic.es (A.G.); celvira@ictp.csic.es (C.E.); jrodriguez@ictp.csic.es (J.R.-H.)

**Keywords:** covalent adaptable networks, vitrimers, transesterification, photopolymerization, beta-amino esters, reprocessing

## Abstract

In recent years, efforts have focused on developing repairable, malleable, and recyclable thermoset materials to reduce the growing volume of polymer waste and extend the lifetime of existing polymeric materials. Specifically, associative covalent adaptable networks (CANs), also known as vitrimers, have received considerable attention. In this work, photopolymerizable vitrimers were prepared by combining crosslinkers containing *β*-amino esters in their structure with hydroxylated acrylate or methacrylate monomers, with the aim of reprocessing these materials through the activation of transesterification reactions. The network design and photopolymerization conditions were optimized to ensure the successful formation of the vitrimers. Tunable mechanical and thermal properties were achieved by varying their chemical composition. Furthermore, the reprocessing ability of these materials was confirmed through thermal treatments. Additionally, these vitrimers exhibited the ability to undergo hydrolysis in basic aqueous media, providing an alternative pathway for recycling.

## 1. Introduction

The increasing volume of polymer waste produced requires the development of more effective strategies aimed at minimizing waste generation, enhancing plastic disposal, and promoting both recycling and reuse [1,2]. In conjunction with these strategies, current research efforts are being made to progress towards a zero-discharge and carbon-neutral scenario [3]. Among others, to reach these two objectives, researchers are developing designs of innovative materials that are recyclable and take into account the entire lifecycle of the plastic material [4]. In this context, several alternatives are currently being explored within the framework of what has been named “eco design”. These approaches include, among others, the development of depolymerization methods aimed at recovering virgin monomers [5], the application of sustainable solvents or use of supercritical fluids [6], the creation of biomass-based polymers [7], or the formulation of crosslinking and decrosslinking strategies [8], to mention just a few of them.

In particular, a major issue currently faced by the industry is the widespread utilization of thermosetting polymers which, according to Morici and Dintcheva [9], account for approximately 12% of the total global plastic production, i.e., around 44 million tons. Thermosets are three-dimensional polymer networks, where the polymer chains are covalently crosslinked. This unique structure renders them ideal for demanding applications that require properties such as creep resistance, solvent resistance, heat resistance, and high mechanical strength. Moreover, thermosetting with improved properties are usually obtained by preparing composites with the incorporation of carbon [10], glass [11], or aramid fibers [12]. As a result, the applications of thermosetting polymers include structural components, coatings, adhesives, electronics, and composites [13]. However, in contrast to thermoplastics, thermosets do not possess the ability to flow, which prevents them from being reshaped, reprocessed, or recycled, consequently resulting in considerable waste generation. More precisely, current waste from thermosets and thermoset composites is typically processed into fillers, incinerated and, in spite of the new ongoing recycling developments, a significant portion ultimately finds its way to landfills [9].

In this context, numerous studies are currently being conducted to merge the flexibility and reprocessability of thermoplastics with the performance benefits of thermosets. In order to overcome the thermoset issues mentioned above, researchers have developed strategies introducing in the thermoset materials crosslinkers that enable them to straightforwardly break the links while retaining their robustness. Two different alternatives have been so far explored separately that involve either the use of degradable crosslinkers or introduce covalent reversible crosslinks [9,14,15].

The dominant approach focuses on creating polymer networks with crosslinks that can be disrupted and reformed within a designated temperature range, activated by external stimuli [13]. This idea leads to the development of covalent adaptable networks (CANs). These networks are three-dimensional structures that incorporate dynamic covalent bonds (DCBs), which can be exchanged through reversible chemical reactions when subjected to external stimuli, allowing for the reconfiguration of the network’s topology [16]. Depending on the nature of the reversible covalent bonds, various stimuli can be used to shift the equilibrium between the associated and dissociated states. For instance, reversible covalent networks have been developed using thermally activated groups (e.g., Diels–Alder adducts, alkoxyamines, urethanes, ureas, triazolinediones, 1,2,3-triazoliums, and aniliniums); photo-activated groups (e.g., coumarin dimers, trithiocarbonates, disulfides, and diarylbibenzofuranone derivatives); and chemically activated groups (e.g., boronic esters, disulfides, acylhydrazones, imines, and acetals) [13]. Moreover, two distinct types of CANs can be defined depending on the mechanism of the reversible exchange reaction of the dynamic covalent bonds. The first ones are dissociative CANs, where the DCB is broken before being re-formed. As a consequence, during the intermediate step the crosslinking density decreases, leading to a decrease in viscosity. Representative examples of dissociative dynamic covalent bond exchanges include Diels–Alder cycloadditions, thiol-/aza-Michael addition, boronic ester cycloaddition, and silanol bonds [17]. The second ones are associative CANs, also known as vitrimers, first introduced by Leibler and co-workers in 2011 [18]. In this case, the DCB is not broken until a new bond is formed, thus the cross-linking density remains constant. As a result, dynamic polymer networks based on associative bonds exhibit higher thermal stability, enhanced solvent resistance and improved resistance to material deformation compared to their dissociative counterparts. Typical examples of associative exchange interactions include transesterification, transamination, and disulfide metathesis [17].

In particular, vitrimers based on dynamic transesterification reactions between ester bonds and –OH groups have been the subject of extensive investigation, as this mechanism can be applied to many commercially available thermosetting polymer systems. Moreover, the relatively high reaction temperature (>120 °C) of dynamic transesterification reactions enables the development of rigid polymer materials with high service temperatures [19]. Recently, this mechanism has been employed, for instance, to prepare vitrimerized thermoplastics, including polylactic acid (PLA) [20], polybutylene terephthalate (PBT) [21], and polyethylene (PE) [22]. These studies expand the vitrimer concept into the realm of traditional thermoplastics, opening up new possibilities for broader applications. Since Leibler et al. [18] introduced the first repairable and malleable crosslinked polymer material based on dynamic transesterification reactions in 2011, numerous related publications and patents have been reported [19,23].

Herein, we focus on the design of crosslinked networks based on built-in recyclability bearing simultaneously dynamic links, designed to be recycled by reprocessing (CANs) and degradable links thus offering the possibility to recycle by formation of linear thermoplastic polymer chains. To the best of our knowledge, there is no precedent in the literature of a system combining both reprocessing alternatives.

As will be thoroughly described, we propose the elaboration of CANs with tunable mechanical and thermal properties based on the photopolymerization of mixtures containing a hydrolysable crosslinker containing *β*-amino esters in its structure and different acrylate/methacrylate monomers. As will be described, for this purpose, crosslinkers were synthesized via aza-Michael addition of several diamines to acrylates. The design of the network was optimized, and vitrimers were prepared through photopolymerization, as it is a rapid and straightforward technique. The reprocessing ability of the materials was evaluated by activating transesterification reactions investigating the role of the catalyst employed during the reprocessing step [17].

## 2. Experimental Part

### 2.1. Materials

2-hydroxyethyl methacrylate (HEMA) (CAS-No: 868-77-9, ≥99%), 2-hydroxy-3-phenoxypropyl acrylate (HPPA) (CAS-No: 16969-10-1, ≥80%), Jeffamine ED-600 (CAS No: 65605-36-9), 4,7,10-trioxa-1,13-tridecanediamine (CAS-No: 4246-51-9, 97%), piperazine (CAS-No: 110-85-0, 99%), acetic acid, zinc acetylacetonate hydrate (CAS-No: 108503-47-5, 99.995%), and diphenyl(2,4,6-trimethylbenzoyl) phosphine oxide (TPO) (CAS-No: 75980-60-8, 97%) were purchased from Sigma-Aldrich. 2-hydroxyethyl acrylate (HEA) (CAS-No: 818-61-1, >95%) and 3-(acryloyloxy)-2-hydroxypropyl methacrylate (CAS-No: 1709-71-3, >80%) (AHPMA) were purchased from TCI.

Dichloromethane and hexane were purchased from Scharlab (Madrid, Spain) and employed as received. Powdered silicon sheets were purchased from Arpival S.A. (Madrid, Spain) and polystyrene sheets were purchased from Resopal (Madrid, Spain).

### 2.2. Methods

Attenuated total reflectance-infrared spectroscopy (ATR-FTIR) was performed using a FT-IR Spectrometer (Spectrum Two, Perkin Elmer Massachusetts, Waltham, MA, USA) from PerkinElmer, equipped with a diamond crystal ATR accessory. Spectra were recorded with a resolution of 4 cm^−1^ in the range of 4000–450 cm^−1^ for each sample.

^1^H NMR spectra, using CDCl_3_ as solvent were recorded on a Varian System 500 spectrometer (Palo Alto, CA, USA) equipped with a 5 mm HCN cold probe with field Z-gradient, operating at 500.13 MHz. This technique was employed for the characterization of the extracts obtained from the photopolymerized materials. For this purpose, the material was immersed in chloroform during 48 h. The material was removed and the solvent evaporated under reduced pressure. The residue was then redissolved in deuterated chloroform and analyzed by ^1^H NMR.

Differential Scanning Calorimetry (DSC) was conducted using a differential scanning calorimeter controlled by METTLER (Greifensee, Switzerland) instrument. Measurements were conducted from −50 °C to 200 °C at a heating rate of 10 °C/min.

Thermogravimetric analyses (TGA) were carried out in a thermogravimetric analyzer controlled by METTLER instrument. Samples were heated from room temperature to 800 °C at a rate of 10 °C/min under a nitrogen atmosphere.

Mechanical properties were evaluated through tensile tests conducted at room temperature. Measurements were performed in a “Universal testing machine”, INSTRON (High Wycombe, UK) brand, equipped with a 100 N load cell, manual jaws, and a test speed of 10 mm/min. For each test, 5 specimens were used, each with a width of 2 mm, a thickness of 0.5 mm, and a length of 35 mm.

The reprocessing of the different vitrimers was carried out by cutting the films obtained by photopolymerization and post-cured into small pieces (1 × 1 cm^2^) and pressed using a Collin (Maitenbeth, Germany) press, where temperatures ranging from 150 °C to 250 °C, pressures between 35 bar and 150 bar, and different time conditions ranging from 5 min to 30 min were applied.

Hydrolysis in basic aqueous media tests were conducted at room temperature. Specimens (1 cm × 1 cm × 0.5 mm) were immersed for up to 7 days in basic aqueous media (20 mg/mL D_2_O/NaOH 1 M). After this period, the solubilization of the specimen was visually analyzed, and ^1^H-NMR analysis was performed to determine the composition of the dissolved fraction.

### 2.3. Monomer Purification

While HEA and HEMA are commercially available with purities ranging from 95% to 99%, HPPA is only available with purities below 80%. In-depth NMR analysis and an exact mass analysis of the commercial HPPA revealed a diacrylate derivative as the main side product, which could participate in subsequent reactions, leading to additional non-reversible crosslinking. Therefore, purification was carried before using this monomer by chromatography, using dichloromethane and hexane as solvents, starting with a 3:1 ratio and finishing with 5:1 to eliminate the diacrylate derivative (see Supporting Information Figures S1–S5).

### 2.4. Synthetic Strategy for the Preparation of the Crosslinkers

The crosslinkers were prepared via aza-Michael addition, mixing 3-(acryloyloxy)-2-hydroxypropyl methacrylate (AHPMA) with different diamine precursors in a 2:1 molar ratio. Specifically, 4,7,10-trioxa-1,13-tridecanediamine, Jeffamine ED-600, and piperazine were used to form TTDA-CL, JA-CL, and PA-CL, respectively. The reaction, based on the regioselective attack of the amine group on the acrylate unit, was carried out overnight at 37 °C in the presence of a catalytic amount of acetic acid to render quantitatively the desired crosslinker. ^1^H NMR of the crosslinkers are included in the Supporting Information (Figures S6–S8).

#### 2.4.1. Preparation of Vitrimers by Photopolymerization

For the preparation of vitrimers, initially, one of the monomers (HEA, HEMA, or HPPA) was mixed with one of the crosslinkers (TTDA-CL, JA-CL or PA-CL) in different weight ratios. Then, TPO, which acts as a photoinitiator (2% by weight relative to the total weight of the monomer and crosslinker), was added, and the mixture was stirred. Next, it was injected into a mold consisting of two polystyrene layers covered with a polyethylene film, and a silicon spacer between the layers, to set the thickness of the sample. Finally, it was placed in a UV chamber equipped with five lamps and a power of 8 W, acquired from VILBER (model BLX-365, Eberhardzell, Germany). After testing various conditions for the photopolymerization, the selected ones were photocuring at room temperature with a wavelength of 365 nm for 40 min, using a 0.5 mm silicon spacer.

#### 2.4.2. Post-Curing of the Photopolymerized Vitrimers

To ensure the complete conversion of the monomer, a post-curing process was carried out after photopolymerization. In all cases, it was performed in a UV chamber Form Cure (model FH-CU-01) at 60 °C, with a wavelength of 405 nm for 30 min. Only for the VitHEMA_9_: PA-CL_1_ the conditions were slightly modified, and the post-curing step was carried out at 70 °C for 45 min.

## 3. Results and Discussion

### 3.1. Design of the Vitrimer Formulation

Herein, we describe the preparation of a series of vitrimers in which both the monomer and the structure of the crosslinker were varied. For this purpose, three different monomers, i.e., 2-hydroxyethyl acrylate (HEA), 2-hydroxyethyl methacrylate (HEMA), and 2-hydroxy-3-phenoxypropyl acrylate (HPPA) were selected based on the gradual variation in the Tg of the resulting homopolymers prepared from each monomer. Specifically, HEA leads to homopolymers with a Tg of 14 °C, HPPA to homopolymers with a Tg of approximately 33 °C, and HEMA to homopolymers with a Tg of around 95 °C. In addition to the expected changes in Tg of the resulting material, these monomers were also chosen as they contain –OH side functional groups, which will enable transesterification, and acrylate or methacrylate groups (in order to carry out the radical photopolymerization).

**Scheme 1:**
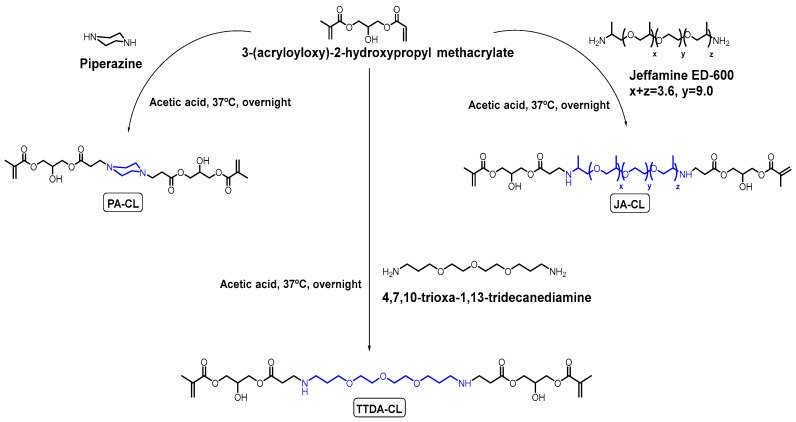
Synthetic strategy for the preparation of the crosslinkers by Michael addition using three different diamines (i.e., piperazine, jeffamine, and 4,7,10-trioxa-1,13-tridecanediamine) and AHPMA as non-symmetric divinyl agent.

In addition to the monomers selected, different dimethacrylates were synthesized and employed as crosslinking agents. For this purpose, three different diamines (see Scheme 1) were employed to react with an asymmetric acrylic structure, i.e., 3-(acryloyloxy)-2-hydroxypropyl methacrylate (AHPMA) containing acrylate and methacrylate groups. As has been previously described [24,25,26], the Michael addition with this asymmetric structure is highly selective towards the acrylate group. Therefore, the three crosslinkers were directly prepared by Michael addition of AHPMA with three selected diamines, i.e., 4,7,10-trioxa-1,13-tridecanediamine (TTDA), Jeffamine ED-600 (JA), and piperazine (PA) in a 2:1 molar ratio. As will be analyzed later, the crosslinker length is expected to play a critical role in the rigidity of the final vitrimer. The reactions were allowed to proceed overnight at 37 °C in the presence of a catalytic amount of acetic acid. In all cases, independently of the diamine employed, as observed by ^1^H NMR (see Supporting Information Figures S6–S8), the reactions proceeded quantitatively without the use of any solvent, and the products formed were used without further purification for the preparation of the covalent adaptable networks [26]. The final product was recovered by selective evaporation of the acetic acid under reduced pressure. The structures of the resulting crosslinkers are shown in Scheme 1.

It is also important to highlight two additional characteristics of these tailor-made crosslinkers. On the one hand, the dimethacrylates are designed to contain *β*-amino ester groups in the main chain. As will be discussed in detail, the *β*-amino esters, known for their high activation towards hydrolysis, are critical to control the degradation of the covalent network and formation of linear chains [27,28]. On the other hand, both monomers and crosslinkers contain –OH side chain functional groups, thus enabling transesterification reactions to occur at the appropriate temperature range and providing the reprocessability required for the material. More importantly, the *β*-amino esters are activated for the transesterification, thus enabling, in some cases, the reaction to occur without the use of catalysts.

### 3.2. Preparation of the Vitrimers by Photopolymerization

**Scheme 2:**
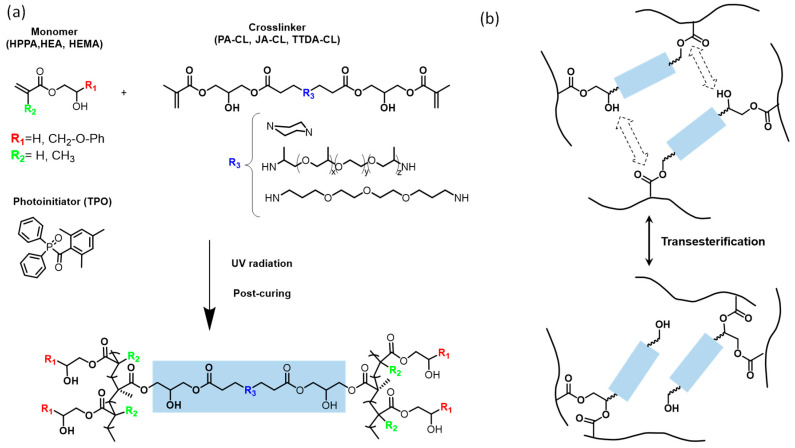
(**a**) Synthetic route to obtain the vitrimers using HPPA, HEA, and HEMA as monomers and PA-CL, JA-CL, or TTDA-CL as crosslinking agents. The reaction was allowed to proceed during 40 min. at room temperature using a UV light (365 nm). (**b**) Thermo-reversible bond exchange through transesterification.

The final vitrimers were obtained upon photopolymerization of a photosensitive mixture comprising both a monomer and a crosslinking agent and using TPO as photoinitiator as it is illustrated in Scheme 2. The photopolymerization reaction was carried out in a UV chamber at room temperature for 40 min with a wavelength of 365 nm. To obtain films with controlled thicknesses, a silicon spacer of 0.5 mm was employed (Figure S9). Finally, a post-curing step was conducted at higher temperatures (60–70 °C depending on the monomer employed) to ensure the complete reaction of the monomers and crosslinking agents, thus avoiding the presence of residual meth/acrylates in the network.

The fabrication of the vitrimers was performed using different weight proportions of the selected monomers and crosslinkers (2:1, 5:1 and 9:1). In contrast, the amount of photoinitiator was kept constant at 2 wt.% relative to the total weight of monomer and crosslinker in all cases (Table 1) [29].

**Figure 1:**
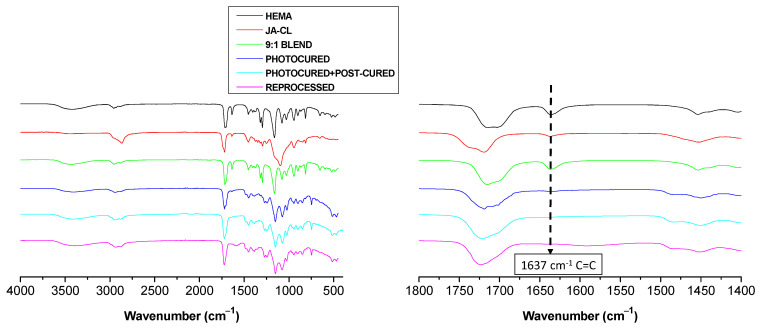
Illustrative FTIR spectra of the vitrimer precursors, i.e., HEMA (black line) and JA-CL (red line), along with a 9:1 blend (green line). In addition, spectra were recorded directly after photopolymerization (dark blue line), after post-curing (light blue line), and after reprocessing (pink line). On the left, the full spectra are shown, and for clarity purposes, a zoom of the spectra in the region between 1800 cm^−1^ and 1400 cm^−1^ is depicted on the right.

The photopolymerization reaction was monitored by FT-IR. In particular, following the signal at 1637 cm^−1^ which is characteristic of the C=C bond of the vinyl group [30,31]. During the photocuring step, this characteristic band is gradually reduced, indicating the consumption of the monomer during the photocuring reaction (Figure 1). However, it was not until the post-curing process was carried out that the signal almost completely disappeared, demonstrating the importance of this step. In addition to the post-curing step, the role of further reprocessing on the remaining traces of double bond has also been investigated. Interestingly, reprocessing at high temperature favors the chain mobility and allows the polymerization to further proceed. As a result, no signal of double bond was observed upon this additional step (considering the detection limits of the technique).

**Figure 2:**
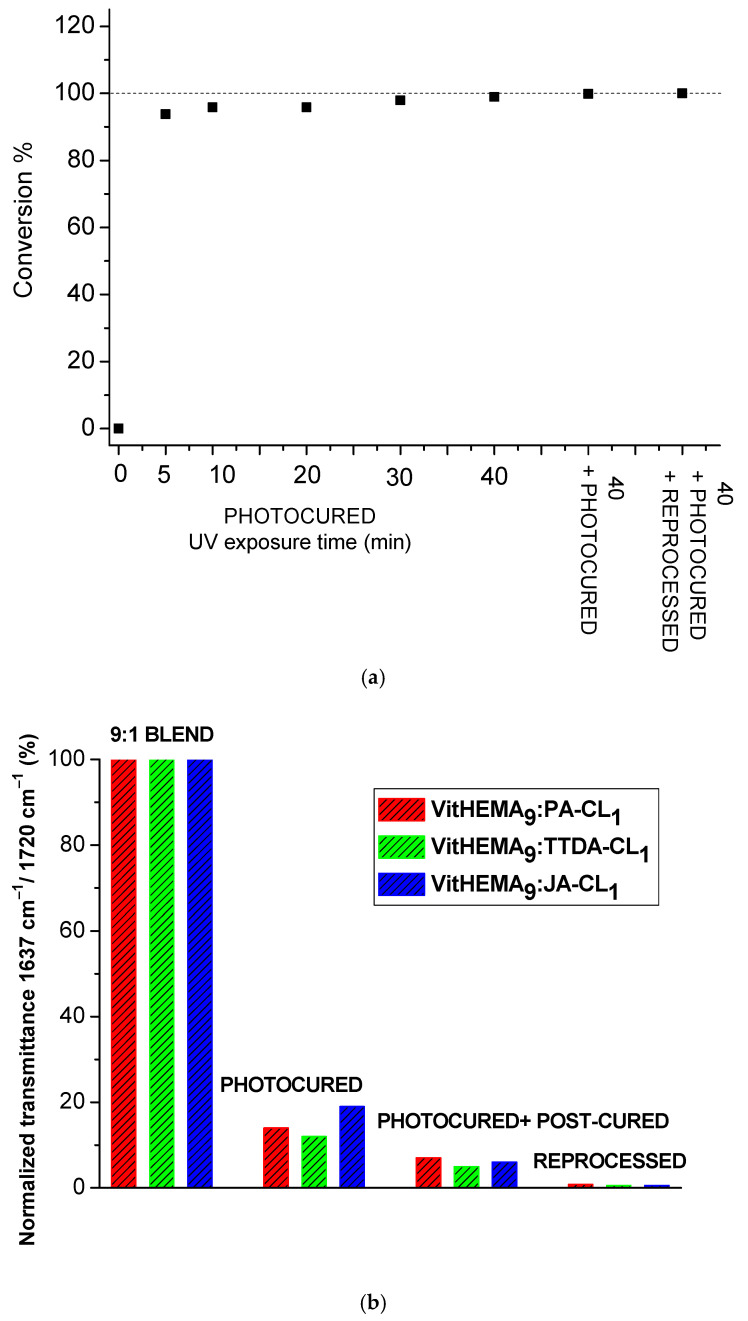

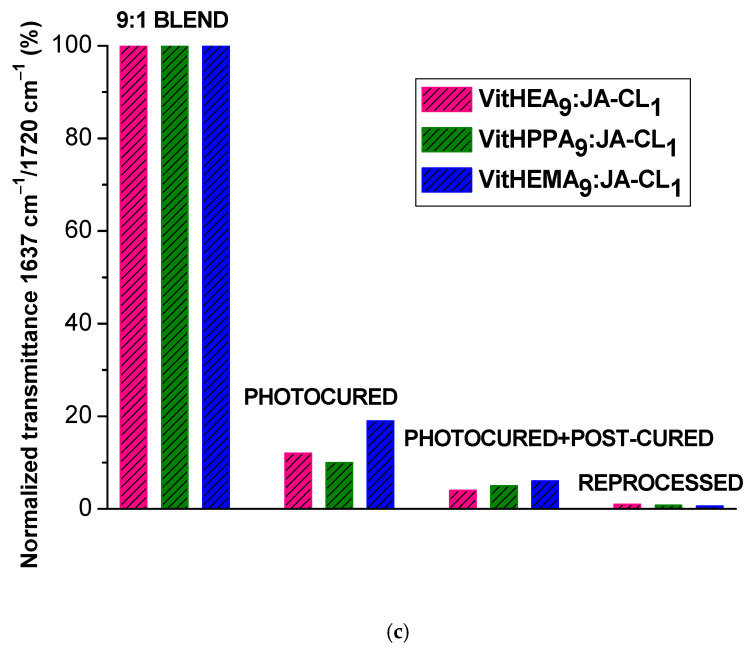
Representation of the normalized transmittance of the characteristic peak of the vinyl group at 1637 cm^−1^ relative to the peak of the carbonyl group at 1720 cm^−1^ in %. (**a**) Conversion vs. time calculated from the FT-IR spectra illustrated for the vitrimer VitHEMA_9_:PA-CL_1_; (**b**) VitHEMA_9_:PA-CL_1_ (red), VitHEMA_9_:TTDA-CL_1_ (light green), and VitHEMA_9_:JA-CL_1_ (blue); (**c**) VitHEA_9_:JA-CL_1_ (pink), VitHPPA_9_:JA-CL_1_ (dark green), and VitHEMA_9_:JA-CL_1_ (blue).

In order to analyze the differences in the photopolymerization step depending on the monomers and crosslinkers employed, in Figure 2 the normalized intensity of the band at 1637 cm^−1^ of the double bond relative to the signal of the carbonyl group at 1720 cm^−1^ for two different series of vitrimers are represented. This representation will enable the comparison of the decrease in the double bond signal and estimate the conversion of the photopolymerization step.

This analysis has been carried out, on the one hand, on a series based on the use of HEMA maintaining a 9:1 monomer-to-crosslinker weight ratio and where the crosslinking agent has been varied (Figure 2a); on the other hand, on a series based on the use of JA-CL as crosslinker and varying the type of monomer employed equally for a monomer-to-crosslinker ratio of 9:1 (Figure 2b). Focusing on Figure 2a we can observe that the photocuring step leads, therefore, to conversions ranging from 80 to 90% (corresponding to a decrease in the signal down to 10–20%). The post-curing step enables the reduction in the remaining double bonds to values between 5 and 10%, indicating conversions of 90–95%. Finally, a processing step (discussed later in this manuscript) leads to materials in which the peak of the double bond cannot be seen (i.e., if it exists, the signal is below the detection limit of the technique) indicating the complete conversion of the acrylates/methacrylates. Although the tendency is similar for the two series included in Figure 2, it is worth noting that in the case of HEMA (which produces vitrimers with higher Tgs), the photocuring step evidenced the lowest conversions of the three monomers employed. These slight differences are reduced in the post-curing step and disappear upon processing (160 °C, 5 min) were all the systems presented quantitative conversions.

These findings were further supported by the analysis of the extracts obtained from these materials. For this purpose, the material was immersed in chloroform during 48 h (see Section 2) to extract residual unreacted monomer. As depicted in Figure S10 (Supporting Information), ^1^H NMR of neither the photocured nor the photocured and post-cured samples presented signals in the range of 5.75–6.5 due to the double bonds.

### 3.3. Thermal Behavior of the Vitrimers: Evaluation of the Tg and the Degradation Temperature

**Figure 3:**
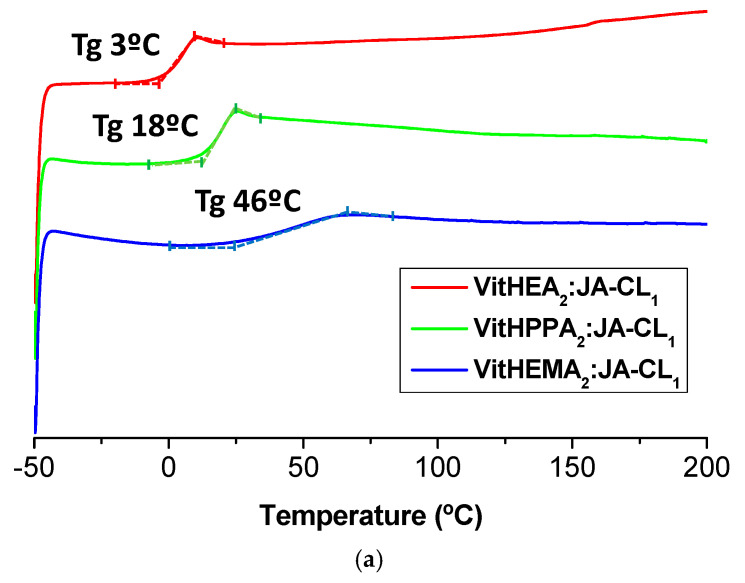

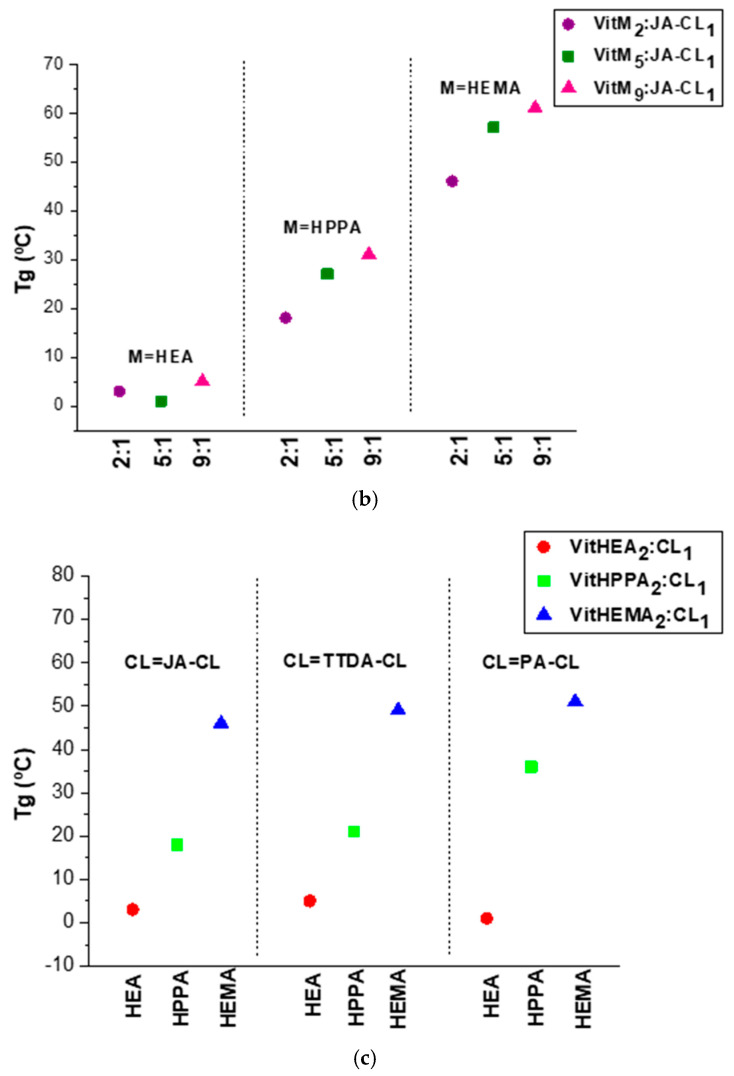
(**a**) DSC traces of the vitrimers prepared using JA-CL as crosslinker and a proportion monomer-to-crosslinker 2:1 for the three monomers employed, i.e., HEA, HPPA and HEMA (**b**) Determination of the Tg for different samples, varying both the monomer and the crosslinker used. (**c**) Comparison of the Tg for samples with different monomer: crosslinker proportions.

The thermal behavior of the vitrimers prepared was analyzed both by DSC and TGA (see Table 2). First of all, DSC was employed to determine the thermal transitions of each sample, in particular, to analyze the differences resulting in Tg due to changes both in chemical composition and proportion of monomer-to-crosslinker. From the DSC experiments the glass transition temperature (Tg) of each sample was determined using the inflection point (see Figure 3a). It is worth mentioning that the degree of crosslinking remains rather low so that the Tg can still be observed in all the vitrimers prepared [32]. In Figure 3a the Tg values measured for a series of samples are represented in which the ratio monomer-to-crosslinker [and, therefore the amount of crosslinker (2:1)] as well as the type of crosslinker (JA-CL) were maintained for these series of experiments thus, varying only the type of monomer employed. As expected, considering the Tg of the homopolymers (Figure S16), the vitrimers containing HEA presented the lowest Tg ~2 °C, those vitrimers prepared using HPPA exhibit Tg values ~18 °C and finally those obtained from HEMA presented the highest Tg ~40 °C.

In addition to this finding, two additional features can also be evidenced. On the one hand, the Tg increased as the proportion of the monomer increased. It is worth mentioning that an increase in the amount of monomer is related to a decrease in the crosslinking density. Therefore, it appears that vitrimers with lower crosslinking density presented higher Tg values. This observation, *a priori* unexpected since, generally, the rigidity of the network raises with a higher proportion of crosslinker [32] can have the following explanation. The crosslinking density remains rather low in all the systems explored (see molar compositions in Table 1) so that the rigidification due to the crosslinking plays a minor role. As a result, in this situation of low crosslinking density the Tg of the monomers employed have a major contribution to the final Tg of the material. (see Figure 3b)

While the type of monomer appears to play a critical role in the final Tg observed and as described above, the type of crosslinker also has a significant influence. As depicted in Figure 3c, in all cases the Tg gradually increases with the rigidity of the monomer and the crosslinker employed. The comparison of vitrimers prepared with the same monomer and different crosslinker reveals a clear trend in the variation in Tg. Vitrimers synthesized with PA-CL, exhibit higher Tg values, followed by vitrimers prepared with TTDA-CL, and lower Tg values were obtained for vitrimers prepared with JA-CL.

**Figure 4:**
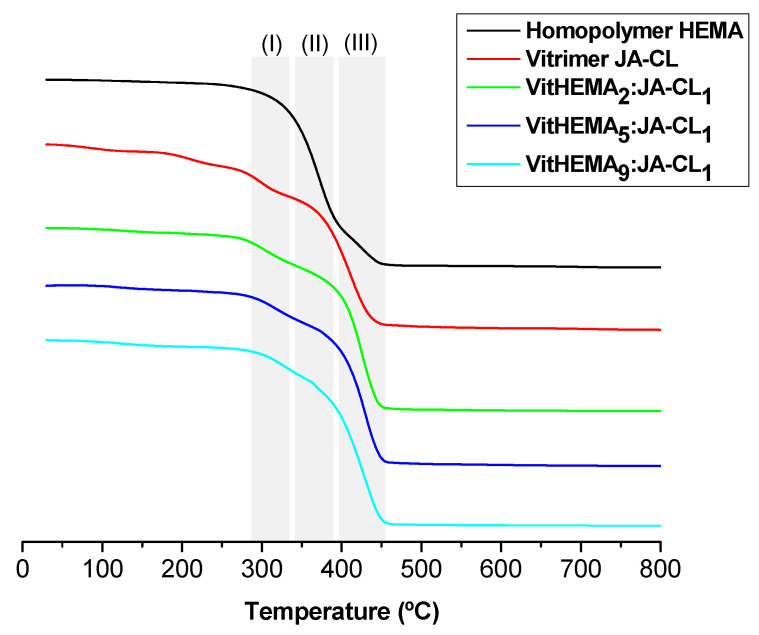
Illustrative thermograms of photopolymerized precursors polyHEMA (black line) and a polymer network prepared exclusively with JA-CL (red line), along with three different samples as follows: VitHEMA_2_:JA-CL_1_ (green line), VitHEMA_5_:JA-CL_1_ (dark blue line), and VitHEMA_9_:JA-CL_1_ (light blue line), showing the effect of varying the monomer-to-crosslinker ratio.

In addition to DSC, TGA was used to establish the range of temperatures to be employed in the reprocessing of the vitrimers in order to avoid eventual degradation. In Figure 4 the TGA of a series of vitrimers prepared from HEMA and JA-CL using different monomer-to-crosslinker ratio (2:1, 5:1 and 9:1) are illustrated. Equally, the TGA of polyHEMA and a crosslinked network prepared using exclusively JA-CL are included. According to the illustrative thermograms presented in Figure 4, three main steps in the thermal decomposition can be observed. The first one occurs between 260 °C and 320 °C and can be attributed to the degradation of the crosslinker in the network. This is supported by the thermograms of the precursors, which indicate that the crosslinker starts to decompose at these temperatures. The second step, observed around 320–380 °C, is associated with the degradation mainly of the polyHEMA segments that according to previous literature produces both chain scission and depolymerization [32,33,34]. Finally, in the range of 400–450 °C both the crosslinker as well as the monomer were completely degraded.

**Figure 5:**
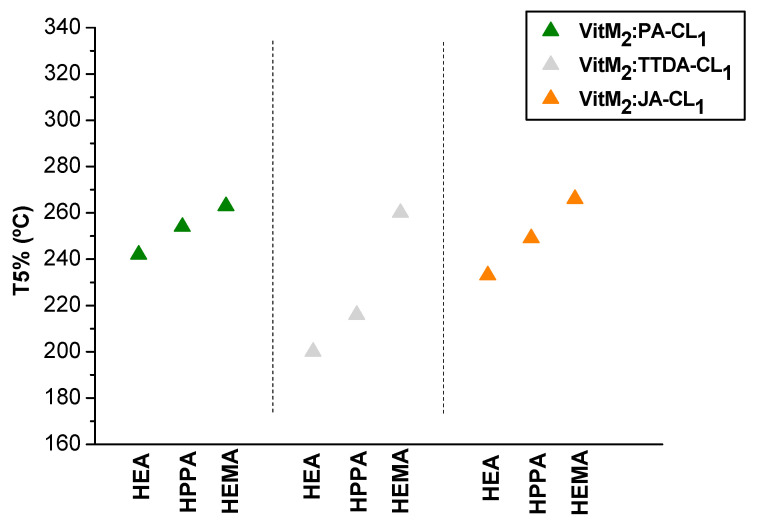
Representation of T_5%_ of the vitrimers prepared with PA-CL (green triangles), TTDA-CL (gray triangles), and JA-CL (orange triangles) samples, comparing thermal stability as a function of the monomer and crosslinker used.

For the selection of the reprocessing conditions of these materials, it is particularly interesting to determine the temperature at which degradation starts. For this purpose, we analyzed the temperature corresponding to a 5% weight loss of the initial mass (T_5%,_) as the reference temperature for reprocessing the samples. This temperature was determined from the thermograms (see Supplementary Information Figure S17) and the values obtained are represented in Figure 5. Independently of the type of crosslinker employed, a general trend can be observed. The T_5%_ measured was higher for those vitrimers prepared with HEMA than for those containing HPPA and lowest values were obtained for vitrimers prepared using HEA. Interestingly, these values follow the same trend as observed in the Tgs of the vitrimers and clearly indicate that the type of monomer appears to be essential for the degradation temperature. Regarding the role of the crosslinking agent, it is possible to conclude from Figure 5 that the vitrimers prepared with TTDA-CL as crosslinker (in particular those with HEA and HPPA) presented a lower degradation temperature starting at 200–220 °C. Vitrimers prepared from crosslinkers PA-CL and JA-CL are more stable and start to degrade at around 240 °C.

### 3.4. Mechanical Properties of the Vitrimers as a Function of the Chemical Composition

**Figure 6:**
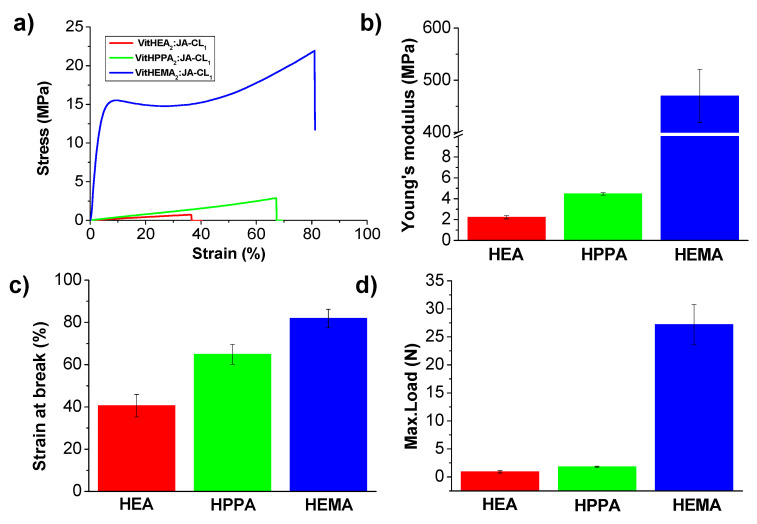
(**a**) Illustrative stress–strain curves obtained for the vitrimers: VitHEA_2_:JA-CL_1_ (red), VitHPPA_2_:JA-CL_1_ (light green), and VitHEMA_2_:JA-CL_1_ (blue). (**b**) Average Young’s modulus. (**c**) Average strain at break. (**d**) Average maximum load.

**Figure 7:**
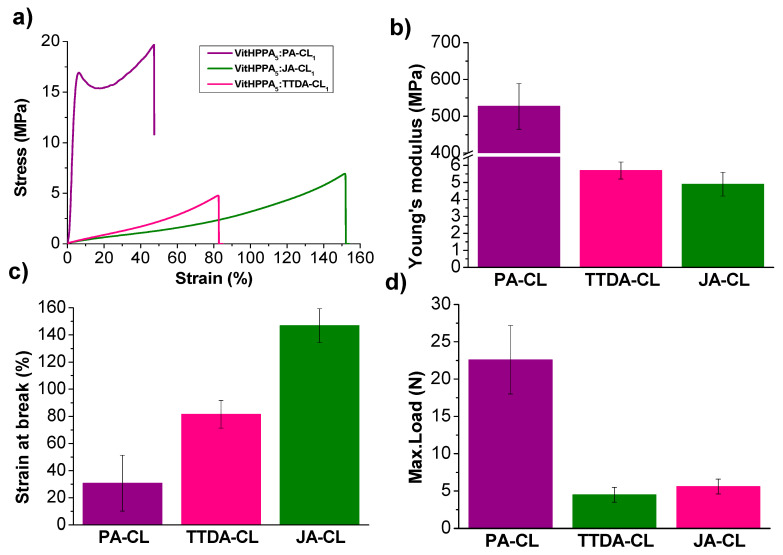
(**a**) Illustrative stress–strain curves of the vitrimers VitHPPA_5_:PA-CL_1_ (purple), VitHPPA_5_:TTDA-CL_1_ (pink) and VitHPPA_5_:JA-CL_1_ (dark green). (**b**) Average Young’s modulus. (**c**) Average strain at break. (**d**) Average maximum load.

**Figure 8:**
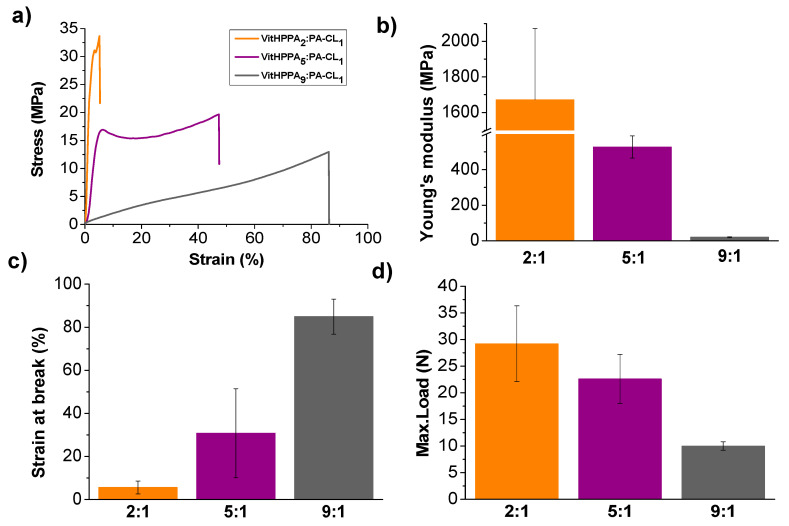
(**a**) Illustrative stress–strain curves of the vitrimers VitHPPA_2_:PA-CL_1_ (orange), VitHPPA_5_:PA-CL_1_ (purple), and VitHPPA_9_:PA-CL_1_ (dark gray). (**b**) Average Young’s modulus. (**c**) Average strain at break. (**d**) Average maximum load.

Another critical aspect of these materials is the evaluation of their mechanical performance. To address this issue, the mechanical properties were evaluated through tensile strength tests conducted at room temperature. The stress–strain curves of the vitrimers along with the corresponding values for the Young’s modulus, strain at break, and maximum load are shown in Figure 6, Figure 7 and Figure 8. Each figure illustrates a series of experiments in which the type of monomer, the crosslinker, and the vitrimer composition are investigated.

Regarding the effect of the monomer on the mechanical properties (Figure 6), the highest Young’s modulus was observed for HEMA, followed by HPPA and HEA. This behavior is directly related to the Tg of the vitrimers. For HEMA, the testing temperature is below its Tg, resulting in low polymer chain mobility and a high modulus (470 MPa). In contrast, for HEA, the testing temperature is above its Tg, leading to high chain mobility and a low modulus (2 MPa). HPPA represents an intermediate case, with a modulus 4.5 MPa, as its Tg is close to room temperature.

Analyzing the stress–strain curves, it can be seen that plastic deformation was reached with HEMA, leading to a higher strain at break (82%) compared to HPPA (65%) and HEA (41%). The maximum load followed a similar trend, being highest for HEMA (27.2 N), followed by HPPA (1.8 N) and lowest for HEA (0.9 N).

To analyze the effect of the crosslinker in Figure 7, the results obtained for vitrimers prepared using HPPA and the three different crosslinkers are represented using, in all the cases, the ratio monomer-to-crosslinker of 5:1. In this case, the highest Young’s modulus was observed with PA-CL (527 MPa), followed by TTDA-CL (6 MPa) and JA-CL (5 MPa). This trend is explained by the fact that a higher crosslinker rigidity results in a higher modulus. Conversely, the strain at break followed the opposite order, reaching 147% with JA-CL, 82% with TTDA-CL, and 31% with PA-CL. This is because greater flexibility is associated with lower crosslinker rigidity. The highest maximum load was achieved with PA-CL (22.6 N), followed by JA-CL (5.6 N) and TTDA-CL (4.5 N).

The last parameter to be analyzed was the effect of changing the monomer:crosslinker proportion. For this purpose, in Figure 8 the mechanical characteristics of a series of vitrimers prepared using HPPA and JA-CL are represented. According to our findings, the Young’s modulus was higher in the case of vitrimers prepared with a 2:1 ratio (1672 MPa), followed by 5:1 (527 MPa) and 9:1 (20 MPa). This trend can be explained taking into account that a higher crosslinker proportion increases the rigidity of the network, leading to a higher modulus. Similarly, the maximum load followed the same tendency, reaching 29.2 N with a 2:1 ratio, 22.6 N with 5:1 and 10 N with 9:1. Finally, as expected, the strain at break (%) showed an inverse trend, being higher with a 9:1 ratio (85%), followed by 5:1 (31%) and 2:1 (6%). This behavior is attributed to the greater molecular mobility and deformation capacity associated with a higher monomer proportion.

### 3.5. Evaluation of the Recyclability of the Vitrimers

In addition to the control of the mechanical properties and composition of the materials, the vitrimers prepared were designed to be recyclable following two alternative routes, i.e., by reprocessing the vitrimers at higher temperatures by transesterification reaction and by hydrolysis of the beta amino esters of the crosslinkers, thus leading to linear polymer chains.

(a) Recyclability of the vitrimers by reprocessing

**Figure 9:**
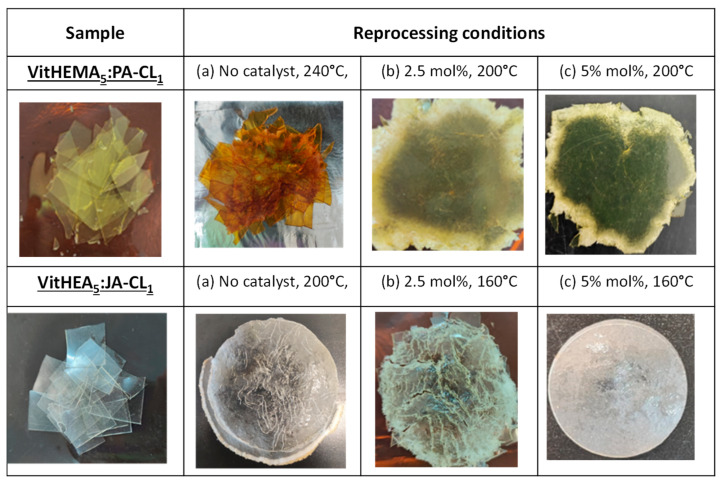
Above: Reprocessing of VitHEMA_5_:PA-CL_1_: (**a**) Without catalyst (**b**) With 2.5 mol% catalyst (**c**) With 5 mol% catalyst. Below: Reprocessing of VitHEA_5_:JA-CL_1_: (**a**) Without catalyst, (**b**) With 2.5 mol% catalyst, (**c**) With 5 mol% catalyst.

On the one hand, the vitrimers were reprocessed taking advantage of the transestererification reactions at temperatures in the range of 160–200 °C maintaining both time (5 min) and pressure (35 bar) constant for all the experiments. The reprocessing temperature was selected based on the Tg of the samples and T_5%,_ which was obtained from the TGA analysis. The photopolymerized films were cut into small pieces (1 cm^2^) and pressed at high temperatures during 5 min. Illustrative images of the films obtained after reprocessing using optimized conditions are included in Figure 9 for vitrimers (monomer/crosslinker ration 5:1) prepared with the highest Tg components, i.e., HEMA and PA-CL (VitHEMA_5_:PA-CL_1_) and vitrimers prepared using the lowest Tg components, i.e., HEA and JA-CL (VitHEA_5_:JA-CL_1_). Finally, it is worth mentioning that a catalyst, zinc acetylacetonate hydrate, has been added to the vitrimer to analyze the processability of the material depending on the amount of activating agent employed. From Figure 9, it can be noted that the addition of a catalyst significantly improves reprocessing. For instance, VitHEMA-PA-CL could not be reprocessed without catalyst even at higher temperatures (~240 °C), where also some degradation as evidenced by the brownish color was observed. Interestingly, in the same vitrimer, the use of 2.5 and 5 wt% clearly improves the reprocessing obtaining films in which the cut pieces are perfectly bonded. In the case of the second series of vitrimers (formed with lower Tg components), the film prepared by reprocessing without catalyst already shows at least partial reprocessability. A decrease in the temperature to 160 °C produced non-reprocessable films, even including 2.5 wt% of catalyst. However, upon increasing the amount up to 5 wt%, the films were reprocessed at the same temperature and maintained a clear color thus evidencing the absence of degradation.

(b) Hydrolysis of the vitrimers and formation of linear polymer chains

**Figure 10:**
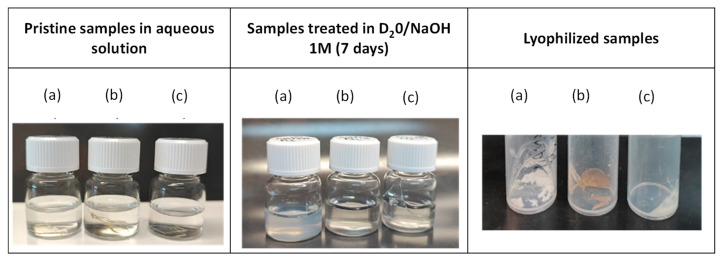
Illustrative examples of the vitrimers partially hydrolyzed to obtain linear polymer chains. The flasks correspond to the following: (**a**) VitHPPA_5_:TTDA-CL_1_, (**b**) VitHEA_5_:JA-CL_1_, (**c**) VitHEMA_5_:PA-CL_1_.

An alternative route to produce alternative reprocessable materials involves the controlled rupture of the crosslinking structure to form linear polymer chains [14,15]. For this purpose, the ability of these materials to undergo hydrolysis in basic aqueous media was also studied. Three different formulations varying both monomer and crosslinker but maintaining the monomer-to-crosslinker ratio (VitHEMA_5_:PA-CL_1_, VitHPPA_5_:TTDA-CL_1,_ VitHEA_5_:JA-CL_1_) were immersed in basic aqueous media (20 mg/mL D_2_O/NaOH 1 M) at room temperature (Figure 10). Although partial hydrolysis of the vitrimers was observed through the first days, a week was employed to complete the hydrolysis. Both VitHPPA_5_:TTDA-CL_1_ and VitHEA_5_:JA-CL_1_ exhibited sensitivity towards hydrolysis in basic media. However, VitHEMA_5_:PA-CL_1_ was not able to solubilize; instead, it transitioned from a rigid material to a partially swollen film, most probably, indicating the presence of residual non-hydrolyzed crosslinks.

**Figure 11:**
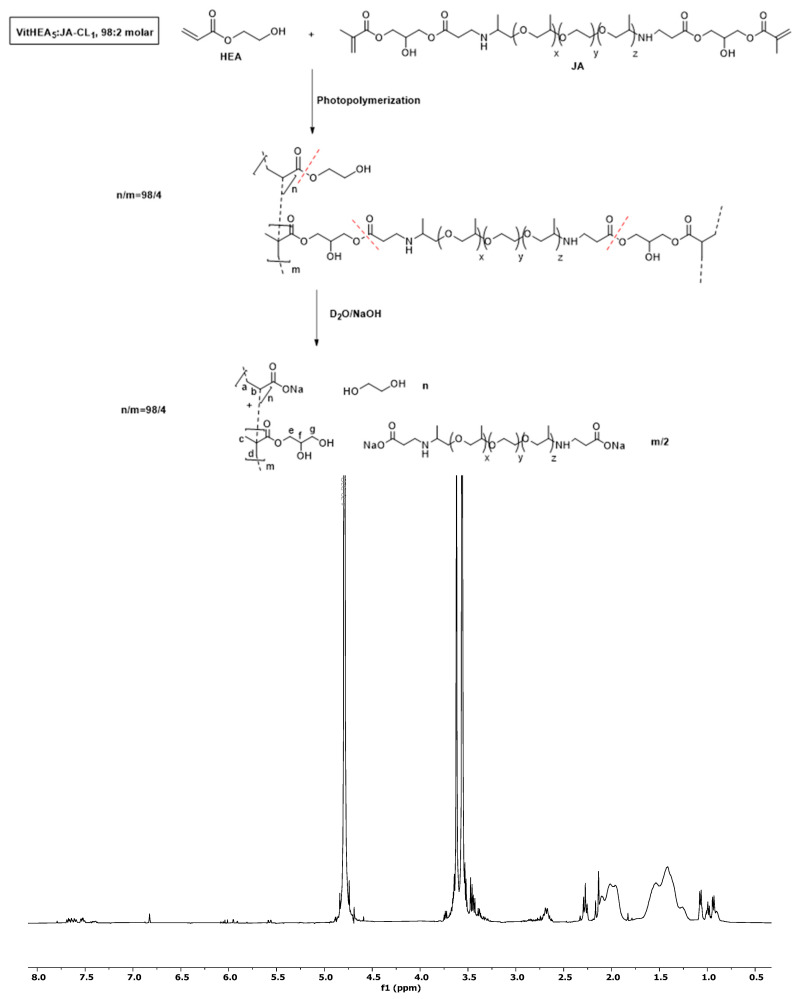
Scheme of the hydrolysis of VitHEA_5_:JA-CL_1_ and ^1^H NMR of the dissolution.

**Figure 12:**
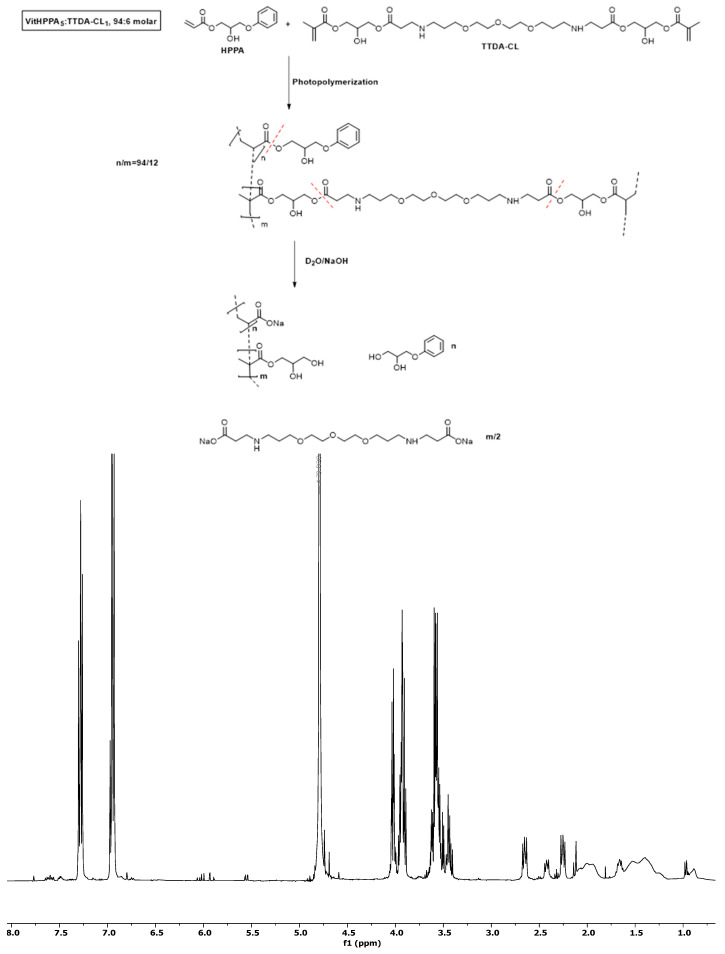
Scheme of the hydrolysis of VitHPPA_5_:TTDA-CL_1_ and ^1^H NMR of the dissolution.

Subsequently, ^1^H NMR analysis was performed on the two completely dissolved vitrimers to determine the hydrolysis mechanism by analyzing the dissolved fractions. In Figure 11 and Figure 12 the proposed hydrolysis mechanism and the ^1^H NMR spectra for the two analyzed systems are included.

The spectrum in Figure 11 is consistent with the proposed degradation mechanism, which includes the hydrolysis of the acrylic ester in these harsh conditions [26]. There is a main polymeric residue rich in sodium acrylate residues. The wide signals centered on 2.0 and 1.4 ppm correspond to the main chain protons of this polymeric residue (CH_2_-CH). The rest of the signals are well defined ones compatible with low molecular weight species produced by the hydrolysis. The singlets centered at 3.55 and 3.65 ppm, which can be assigned to O-CH_2_-CH_2_-O segments of free JA-derived residue and free ethylene glycol, stand out.

The spectrum in Figure 12 is equally compatible with the proposed degradation mechanism. Again, broad signals centered at 2.0 and 1.35 ppm are observed, which can be assigned to a sodium acrylate-rich polymeric residue, as well as a rich set of well-defined signals that are compatible with the presence of low molecular weight compounds. Of particular note are the aromatic signals present in the range 7.3–6.95 ppm, which correspond to the aromatic residue, and the set of signals centered at 3.55 ppm, which can be assigned to the O-CH_2_-CH_2_-O segments of the TTDA-based residue.

## 4. Conclusions

We described the preparation of a novel series of vitrimers able to undergo either reprocessing (transesterification) or hydrolysis to produce further reprocessable materials. The preparation of the vitrimers with different properties (Tg, degradation temperature, and mechanical properties) was carried out through photopolymerization of three different monomers and three different crosslinkers. The crosslinkers were synthesized in one single step by Michael addition using an asymmetric acrylic structure, i.e., 3-(acryloyloxy)-2-hydroxypropyl methacrylate (AHPMA) containing acrylate and methacrylate groups and containing *β*-amino ester groups to ensure the reprocessability. As evidenced by ATR-FTIR, the photopolymerization step requires an additional post-curing step to improve the reaction conversion and a post-processing step to further complete the process.

As a result, a series of vitrimers with Tgs ranging from ~2 °C to ~70 °C and thus presenting an elastic or solid behavior at room temperature could be obtained. In addition, all the vitrimers prepared were stable below 200 °C and started to decompose due to the fragmentation of the crosslinker when temperatures above 220–240 °C were reached.

Finally, in terms of mechanical properties, the combinations of monomers and crosslinking agents enabled the fabrication of vitrimers with a wide myriad of mechanical properties, e.g., vitrimers with elastic modulus ranging from 2 MPa up to 1.6 GPa, strain at break from 20 up to 140% and maximum loads (N) up to 30 N.

The proposed systems were additionally recycled either by thermal treatments or by selective hydrolysis in basic aqueous media; thus, they provide an alternative pathway for recycling. To the best of our knowledge, the systems proposed include, for the first time in their design, the information to enable the two alternative recycling strategies to occur depending on the precise interest.

Ongoing work is currently being carried out to further characterize the transesterification mechanism that, according to our preliminary results, leads to materials with increased modulus and reduced strain resistance during the reprocessing process.

## Figures and Tables

**Table 1:** Composition (weight and mol %) of the series of vitrimers prepared in this study. Monomers: 2-hydroxyethyl acrylate (HEA), 2-hydroxyethyl methacrylate (HEMA) and 2-hydroxy-3-phenoxypropyl acrylate (HPPA). Crosslinkers derived from: 4,7,10-trioxa-1,13-tridecanediamine (TTDA-CL), Jeffamine ED-600 (JA-CL), and piperazine (PA-CL).

Sample Code	Monomer (wt%)	Monomer (Mol%)	Crosslinker (wt%)	Crosslinker (Mol%)	Photoinitiator (wt%)
VitHEA_2_:JA-CL_1_	67	95	33	5	2
VitHEMA_2_:JA-CL_1_	67	94	33	6	2
VitHPPA_2_:JA-CL_1_	67	90	33	10	2
VitHEMA_9_:JA-CL_1_	90	99	10	1	2
VitHEMA_9_:TTDA-CL_1_	90	98	10	2	2
VitHEMA_9_:PA-CL_1_	90	97	10	3	2
VitHPPA_2_:PA-CL_1_	67	82	33	18	2
VitHPPA_5_:PA-CL_1_	83	92	17	8	2
VitHPPA_9_:PA-CL_1_	90	95	10	5	2

**Table 2:** Tg (°C), degradation onset temperature (T_5%_) and Young Modulus (MPa) obtained for the series of vitrimers investigated in this study. Monomers: 2-hydroxyethyl acrylate (HEA), 2-hydroxyethyl methacrylate (HEMA) and 2-hydroxy-3-phenoxypropyl acrylate (HPPA). Crosslinkers derived from: 4,7,10-trioxa-1,13-tridecanediamine (TTDA-CL), Jeffamine ED-600 (JA-CL) and piperazine (PA-CL).

Sample Code	Tg (°C)	Degradation Onset Temperature (T_5%_)	Young’s Modulus (MPa)
VitHEA_2_:JA-CL_1_	3	233	2.3 ± 0.2
VitHEA_5_:JA-CL_1_	1	234	2.2 ± 0.3
VitHEA_9_:JA-CL_1_	5	256	1.3 ± 0.1
VitHPPA_2_:JA-CL_1_	18	249	4.5 ± 0.1
VitHPPA_5_:JA-CL_1_	27	259	4.9 ± 0.7
VitHPPA_9_:JA-CL_1_	31	266	5.1 ± 0.4
VitHEMA_2_:JA-CL_1_	46	266	470 ± 51
VitHEMA_5_:JA-CL_1_	57	277	1072 ± 33
VitHEMA_9_:JA-CL_1_	61	272	1362 ± 156
VitHEA_2_:TTDA-CL_1_	5	200	7.8 ± 0.5
VitHPPA_2_:TTDA-CL_1_	21	216	9.2 ± 0.3
VitHEMA_2_:TTDA-CL_1_	49	260	n.d.
VitHEA_2_:PA-CL_1_	1	242	14.1 ± 1.3
VitHPPA_2_:PA-CL_1_	36	254	1672 ± 401
VitHEMA_2_:PA-CL_1_	51	263	n.d.
VitHPPA_5_:TTDA-CL_1_	25	213	5.7 ± 0.5
VitHPPA_5_:PA-CL_1_	40	259	527 ± 62
VitHPPA_9_:PA-CL_1_	34	192	20 ± 3

## Data Availability

The raw data supporting the conclusions of this article will be made available by the authors on request.

## Supplementary Materials

The following supporting information can be downloaded at: https://www.mdpi.com/article/10.3390/polym17182448/s1, Figure S1. ^1^H NMR of HPPA before purification. Figure S2. ^13^C NMR of HPPA before purification. Figure S3. ^1^H NMR of HPPA after purification. Figure S4. ^13^C NMR of HPPA after purification. Figure S5. ^1^H NMR of the main side product (3-phenoxypropane-1,2-dyil diacrylate). Figure S6. ^1^H NMR of the TTDA-CL by reaction between 4,7,10-trioxa-1,13-tridecanediamine and 3-(acryloyloxy)-2-hydroxypropyl methacrylate (AHPMA). Figure S7. ^1^H NMR of the JA-CL by reaction between Jeffamine ED-600 and AHPMA. Figure S8. ^1^H NMR of the PA-CL by reaction between piperazine and AHPMA. Figure S9. Setup employed for the fabrication of films by photopolymerization. Figure S10. FT-IR spectra of illustrative series of vitrimers to address the success of the photopolymerization and the post-curing steps. On the left, the full spectra are shown, and for clarity purposes, a zoom of the spectra in the region between 1800 cm^−1^ and 1400 cm^−1^ is depicted on the right. In each graph it is shown the spectra of the monomer (black line), the crosslinker (red line), the blend (green line), the vitrimer after photopolymerization (dark blue line) and after post-curing (light blue line): (a_1_) VitHEMA_9_:JA-CL_1_ (a_2_) VitHEMA_9_:TTDA-CL_1_ (a_3_) VitHEMA_9_:PA-CL_1_; (b_1_) VitHEMA_2_:PA-CL_1_ (b_2_) VitHEMA_5_:PA-CL_1_ (b_3_) VitHEMA_9_:PA-CL_1_; (c_1_) VitHPPA_9_:JA-CL_1_ (c_2_) VitHEA_9_:JA-CL_1_ (c_3_) VitHEMA_9_:JA-CL_1_, being (a) Comparison between different crosslinkers, (b) Comparison when changing the monomer:crosslinker proportion and (c) Comparison between different monomers. Figure S11. 1H NMR of: (a) Initial feed employed comprising HEA and the crosslinker based on Jeffamine in a 9:1 ratio. (b) Extract obtained from the photopolymerized sample using chloroform during 48h. (c) Extract obtained from a sample that has been both photopolymerized and post-cured using chloroform during 48 h. Figure S12. Illustrative DSC curves comparing the effect on the glass transition temperature when the monomer varies. Figure S13. Illustrative DSC curves comparing the effect on the glass transition temperature while the crosslinker varies. Figure S14. Illustrative DSC curves comparing the effect of varying the monomer:crosslinker proportion on the glass transition temperature. Figure S15. Illustrative DSC curves comparing the effect on the glass transition temperature when the monomer varies. Figure S16. Glass transition temperature (Tg) values obtained from the DSC curves of the homopolymers formed by each monomer: HEA (red), HEMA (green), and HPPA (blue).
