# Supplementary material for: Design of Vitrimers with Simultaneous Degradable and Dynamic Crosslinkers: Mechanical and Thermal Behavior Based on Transesterification Reactions Between β-Amino Esters and Hydroxylated Acrylate/Methacrylate Monomers
Source: Polymers (Basel). 2025 Sep 10;17(18):2448. doi: 10.3390/polym17182448 (PMC12473400; doi:10.3390/polym17182448)
Supplement: Supplementary file 1 [file polymers-17-02448-s001.zip › polymers-3759110-supplementary.pdf]

Supporting information to accompany

Design of Vitrimers with Simultaneous Degradable and Dynamic Crosslinkers: Mechanical and Thermal Behavior Based on Transesterification Reactions Between β-Amino Esters and Hydroxylated Acrylate/Methacrylate Monomers

Naroa Ayensa[a], Felipe Reviriego[a*], Helmut Reinecke[a*], Alberto Gallardo[a], Carlos Elvira[a], Juan Rodríguez-Hernández[a]

[a]*Instituto de Ciencia y Tecnología de Polímeros (ICTP), CSIC, C/Juan de la Cierva 3, 28006 Madrid, Spain. Email: freviriegop@ictp.csic.es, hreinecke@ictp.csic.es*

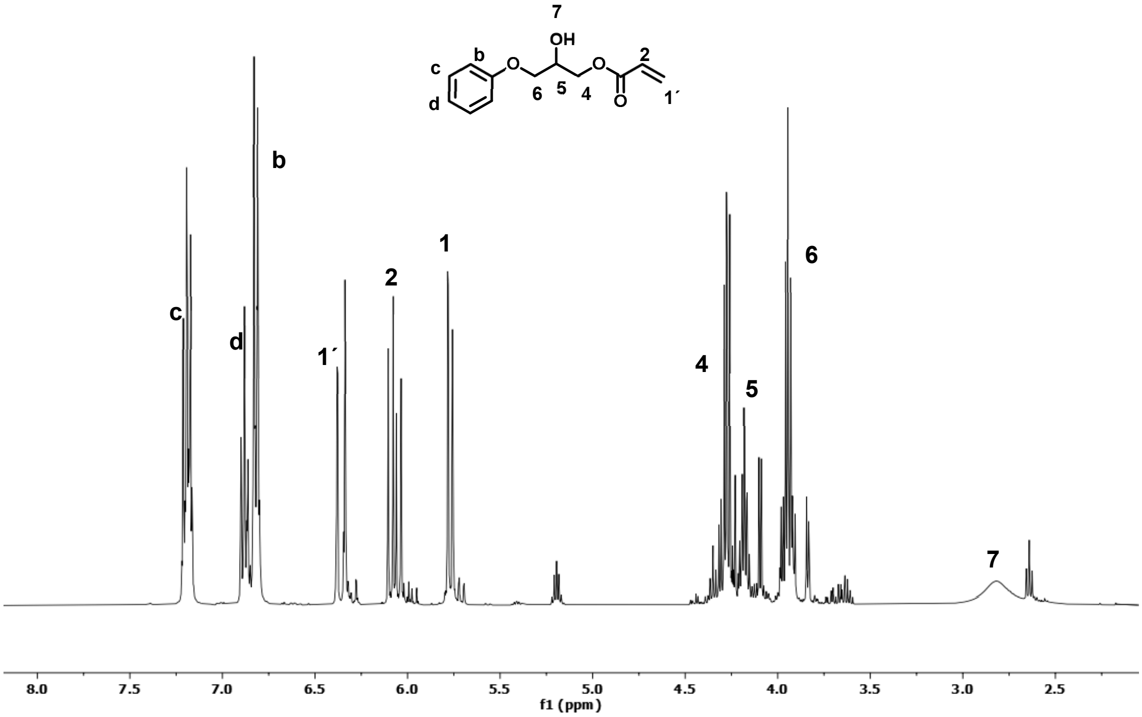

Figure S1. ^{1}H NMR of HPPA before purification.

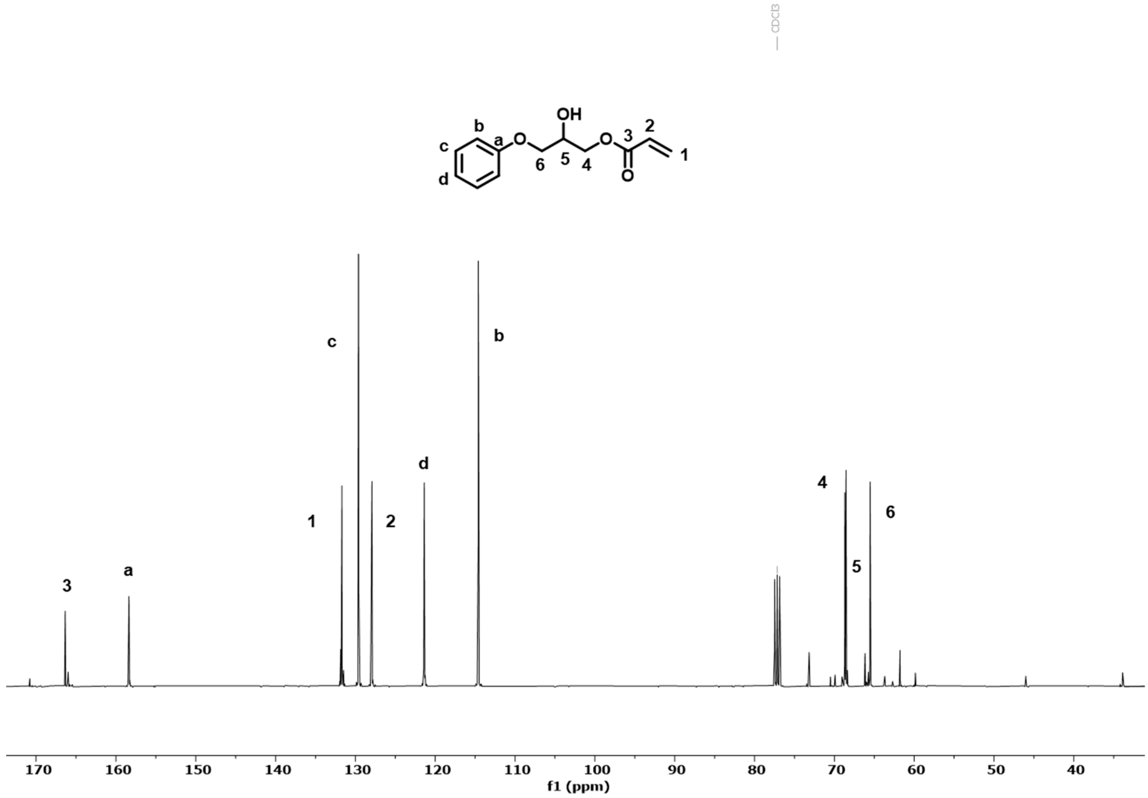

Figure S2. ^{13}C NMR of HPPA before purification.

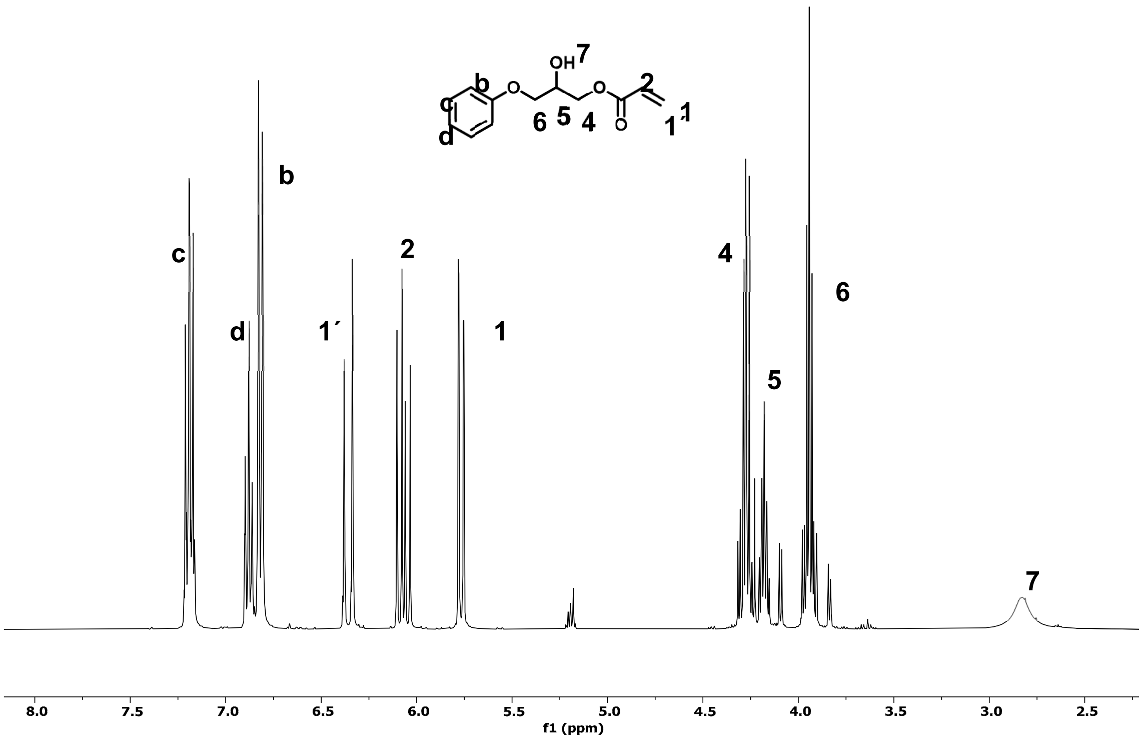

Figure S3. ^{1}H NMR of HPPA after purification.

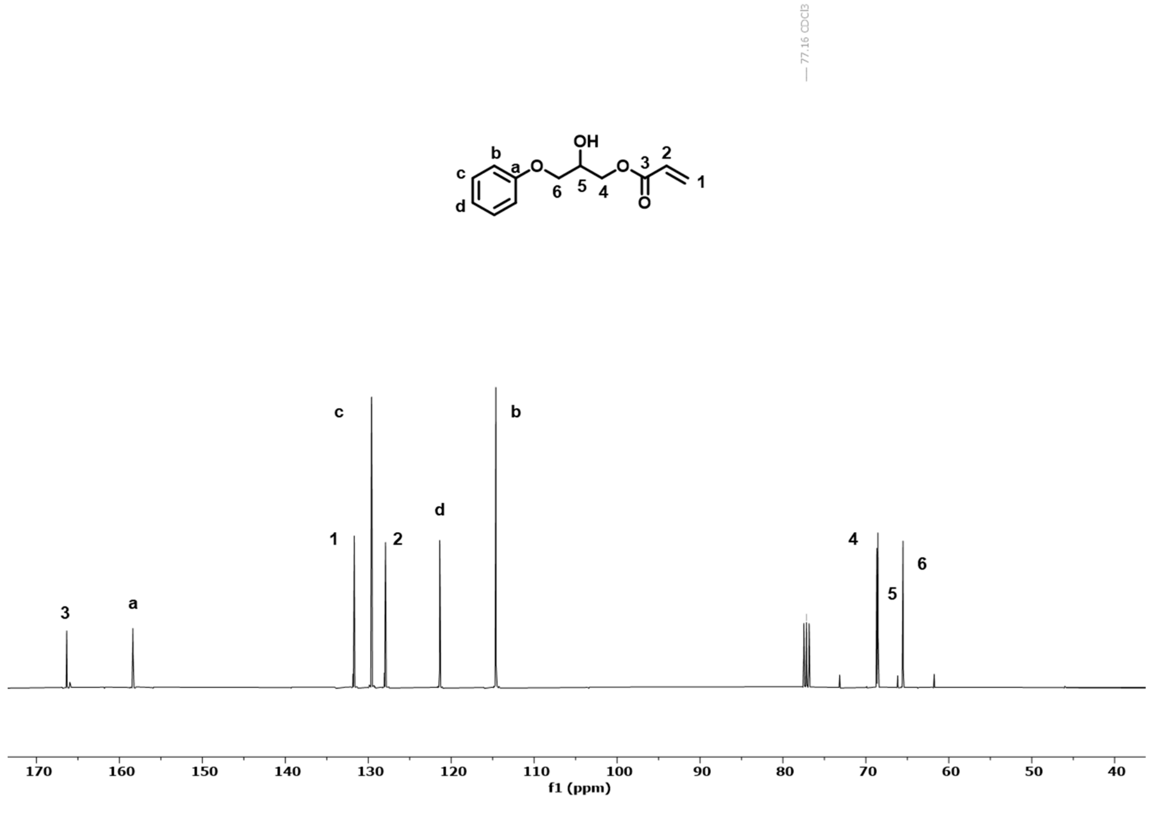

Figure S4. ^{13}C NMR of HPPA after purification.

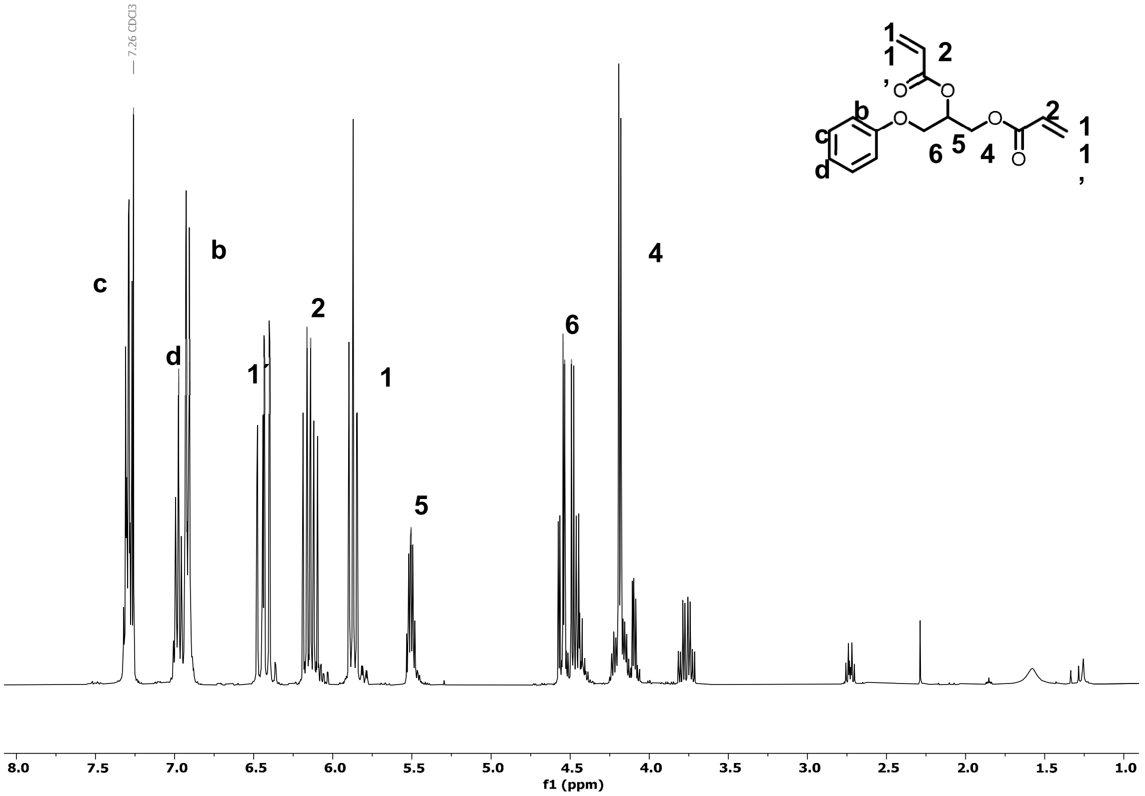

Figure S5. ^{1}H NMR of the main side product (3-phenoxypropane-1,2-dyil diacrylate).

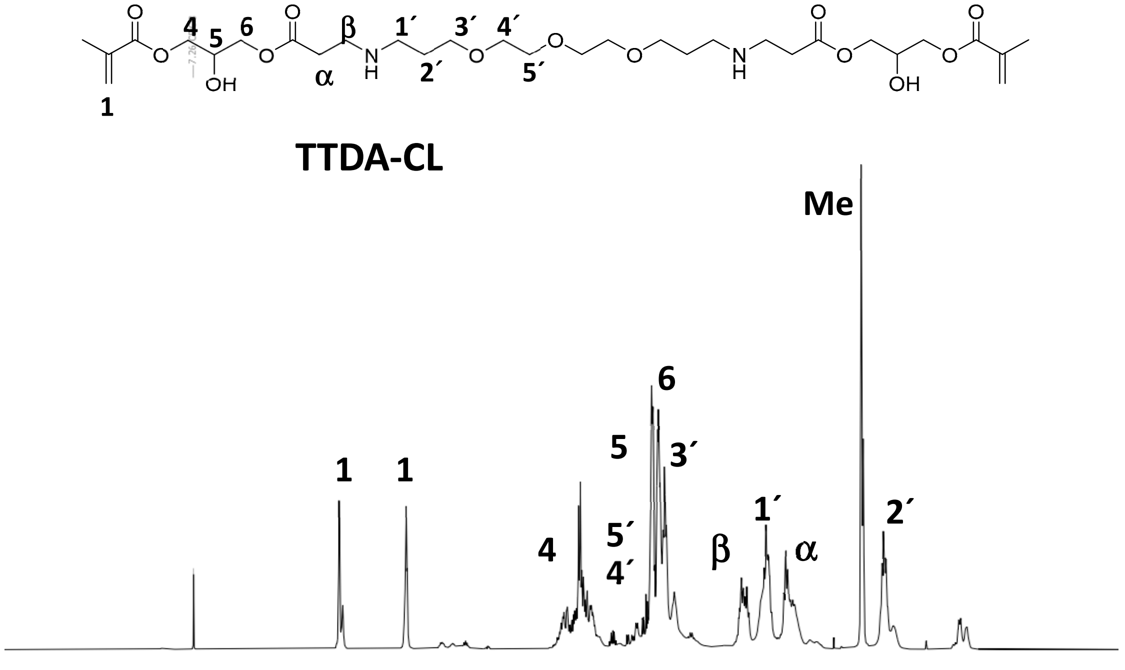

Figure S6. ^{1}H NMR of the TTDA-CL by reaction between 4,7,10-trioxa-1,13-tridecanediamine and 3-(acryloyloxy)-2-hydroxypropyl methacrylate (AHPMA).

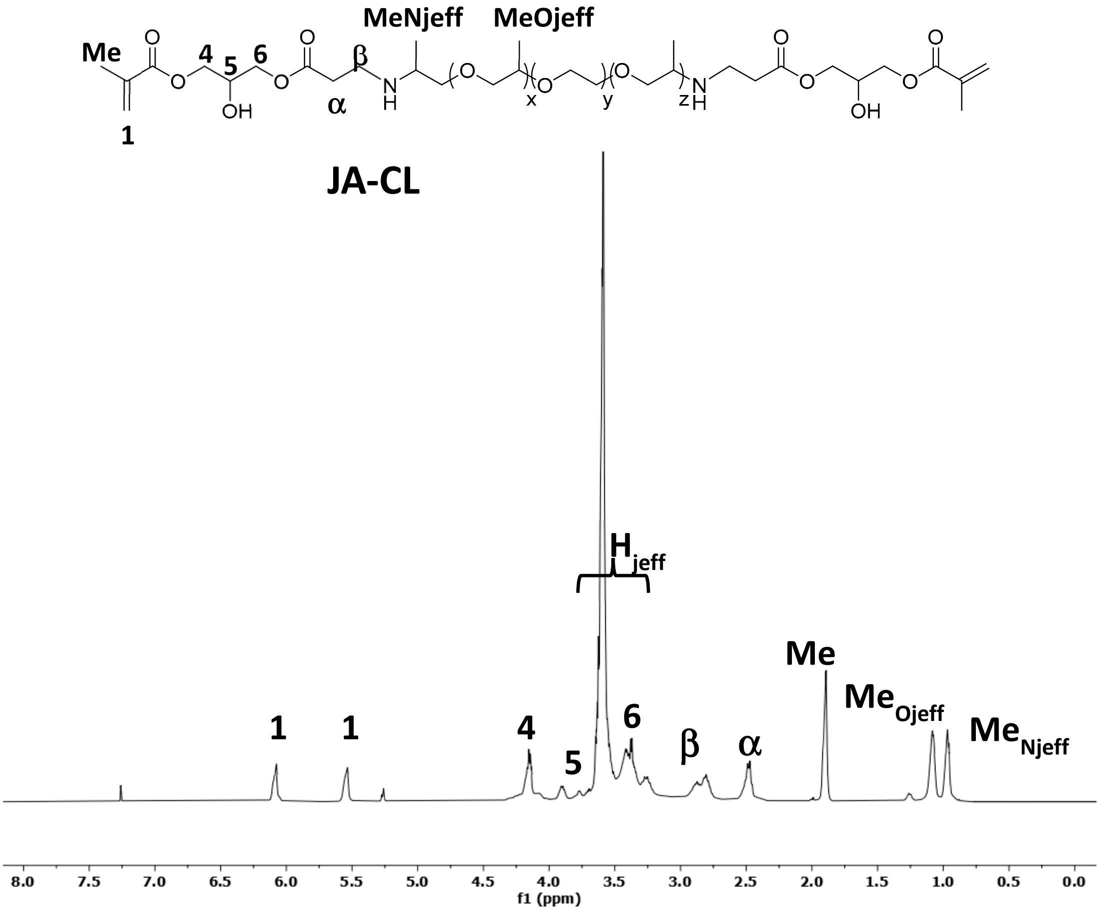

Figure S7. ^{1}H NMR of the JA-CL by reaction between Jeffamine ED-600 and AHPMA.

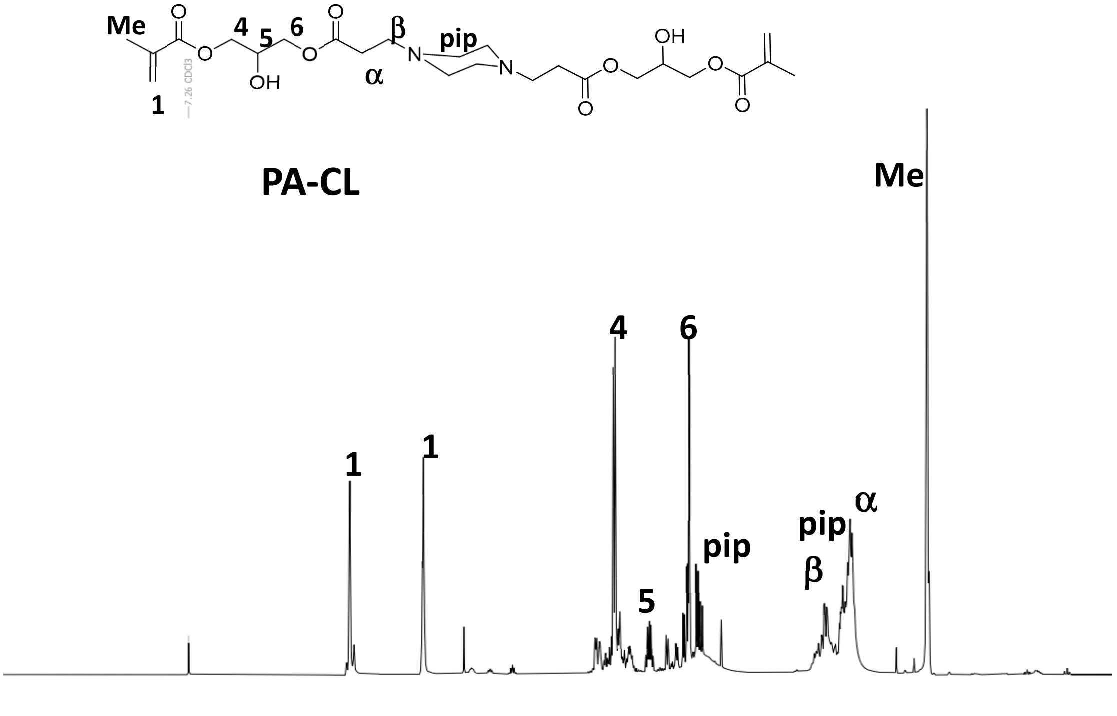

Figure S8. ^{1}H NMR of the PA-CL by reaction between piperazine and AHPMA.

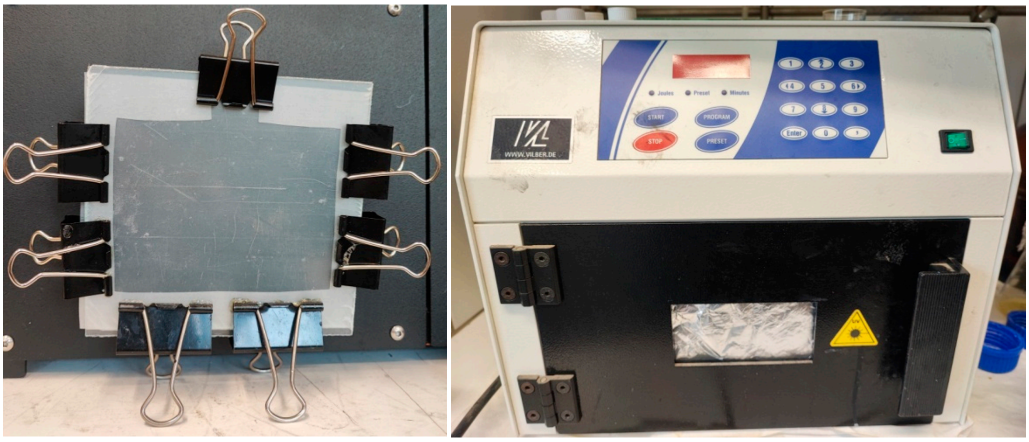

Figure S9. Setup employed for the fabrication of films by photopolymerization.

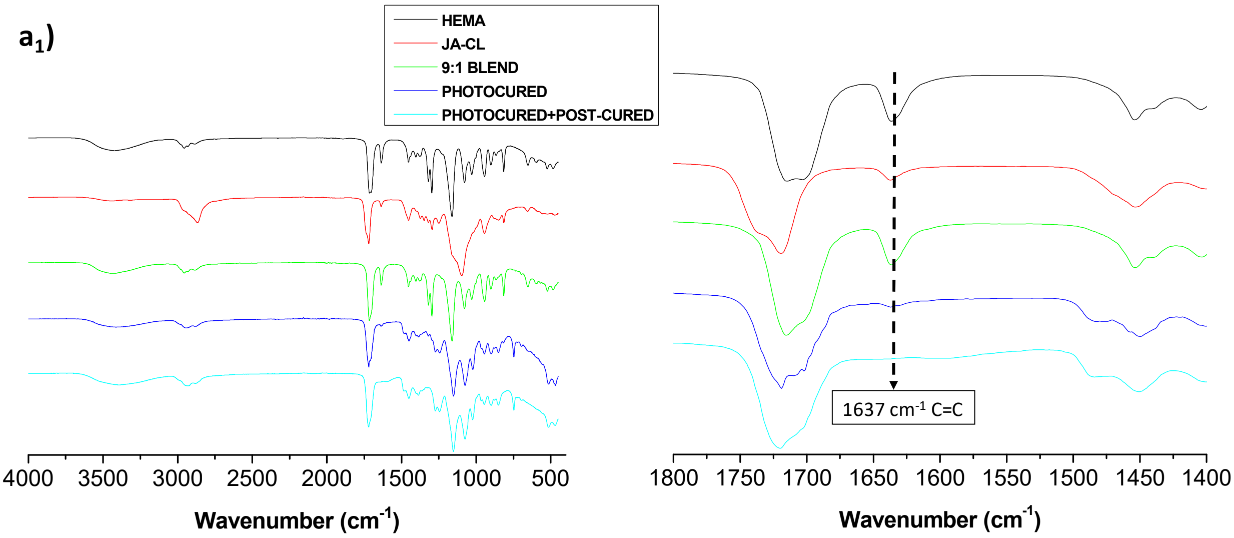

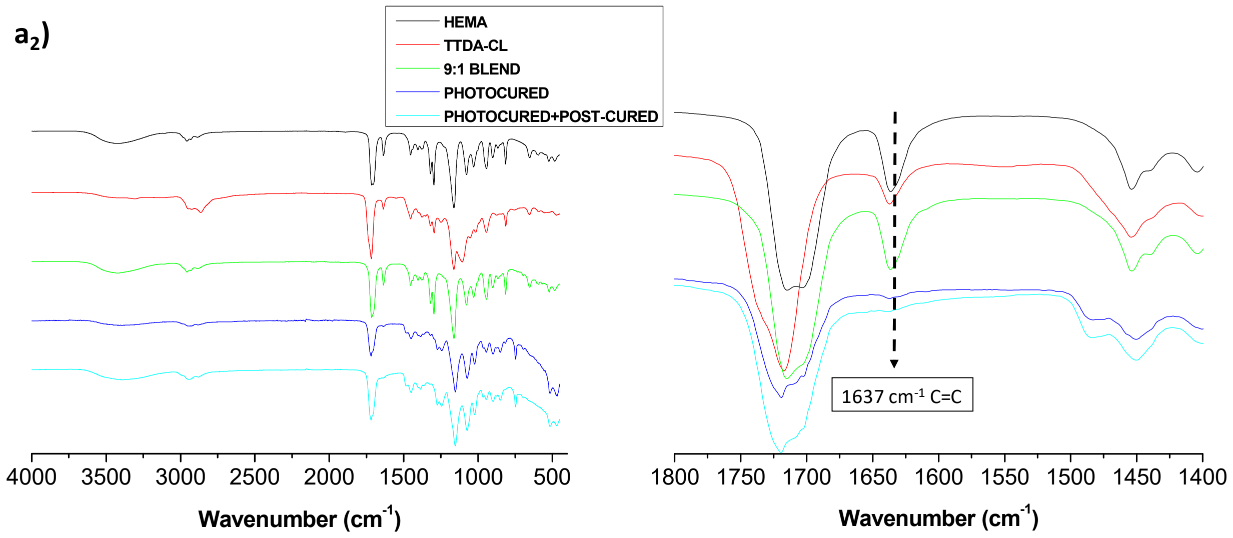
a2)
HEMA
TTDA-CL
9:1 BLEND
PHOTOCURED
PHOTOCURED+POST-CURED
1637 cm-1 C=C
4000 3500 3000 2500 2000 1500 1000 500
Wavenumber (cm-1)
1800 1750 1700 1650 1600 1550 1500 1450 1400
Wavenumber (cm-1)

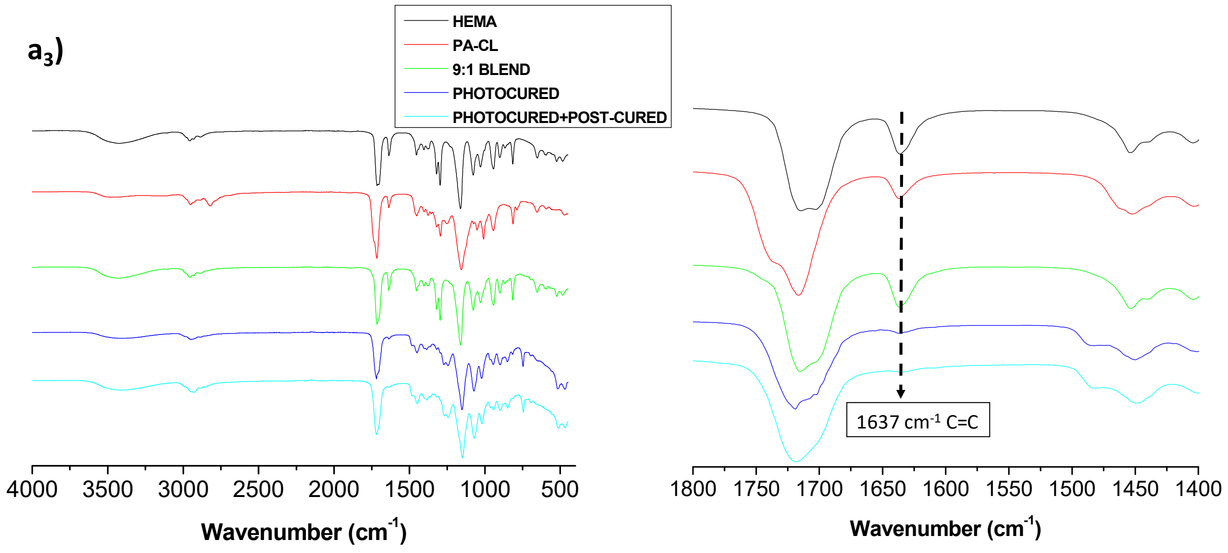
a3)
HEMA
PA-CL
9:1 BLEND
PHOTOCURED
PHOTOCURED+POST-CURED
1637 cm-1 C=C
4000 3500 3000 2500 2000 1500 1000 500
Wavenumber (cm-1)
1800 1750 1700 1650 1600 1550 1500 1450 1400
Wavenumber (cm-1)

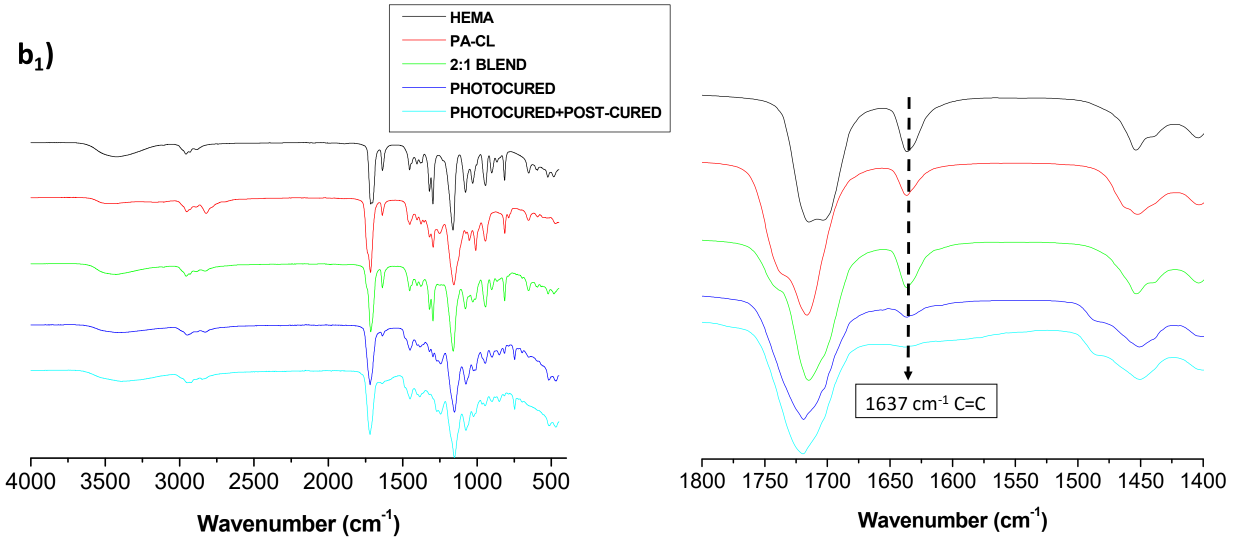
b1)
HEMA
PA-CL
2:1 BLEND
PHOTOCURED
PHOTOCURED+POST-CURED
1637 cm-1 C=C
4000 3500 3000 2500 2000 1500 1000 500
Wavenumber (cm-1)
1800 1750 1700 1650 1600 1550 1500 1450 1400
Wavenumber (cm-1)

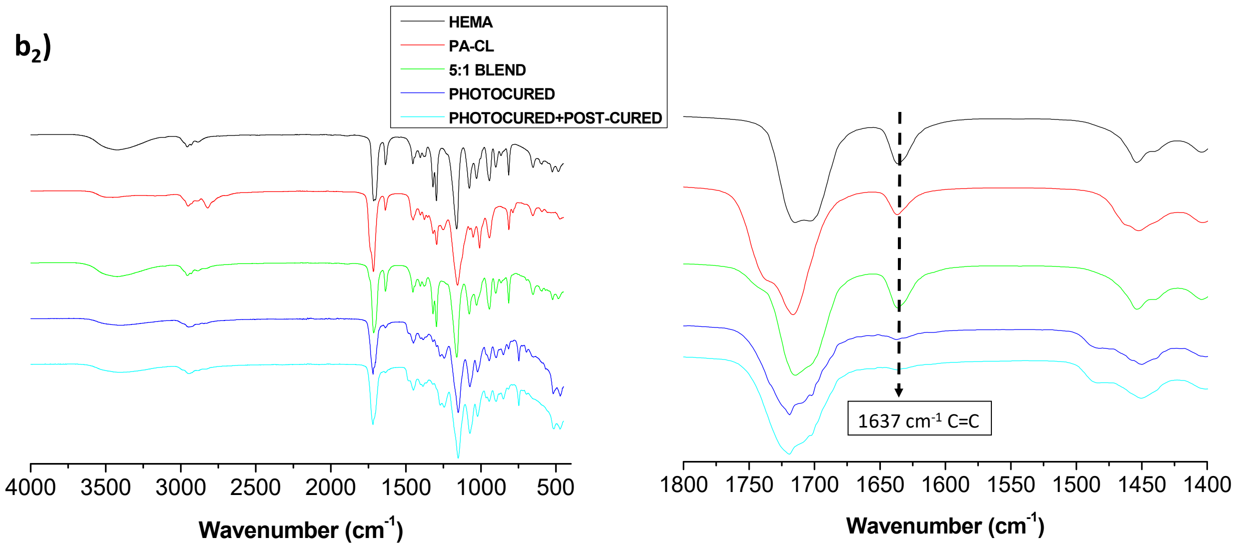
b2)
HEMA
PA-CL
5:1 BLEND
PHOTOCURED
PHOTOCURED+POST-CURED
4000 3500 3000 2500 2000 1500 1000 500
Wavenumber (cm-1)
1637 cm-1 C=C
1800 1750 1700 1650 1600 1550 1500 1450 1400
Wavenumber (cm-1)

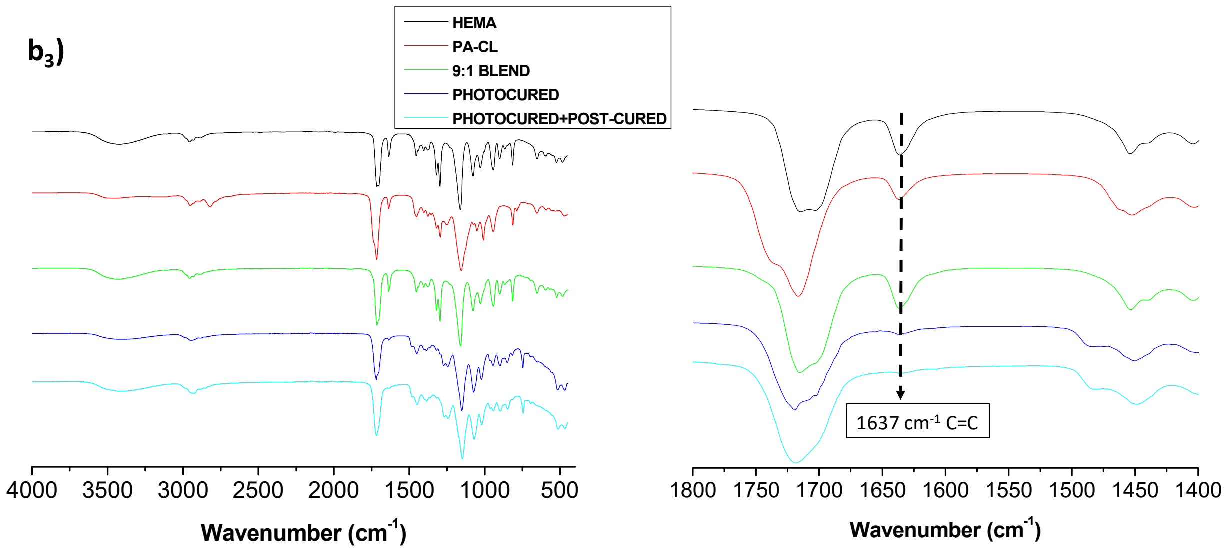
b3)
HEMA
PA-CL
9:1 BLEND
PHOTOCURED
PHOTOCURED+POST-CURED
4000 3500 3000 2500 2000 1500 1000 500
Wavenumber (cm-1)
1637 cm-1 C=C
1800 1750 1700 1650 1600 1550 1500 1450 1400
Wavenumber (cm-1)

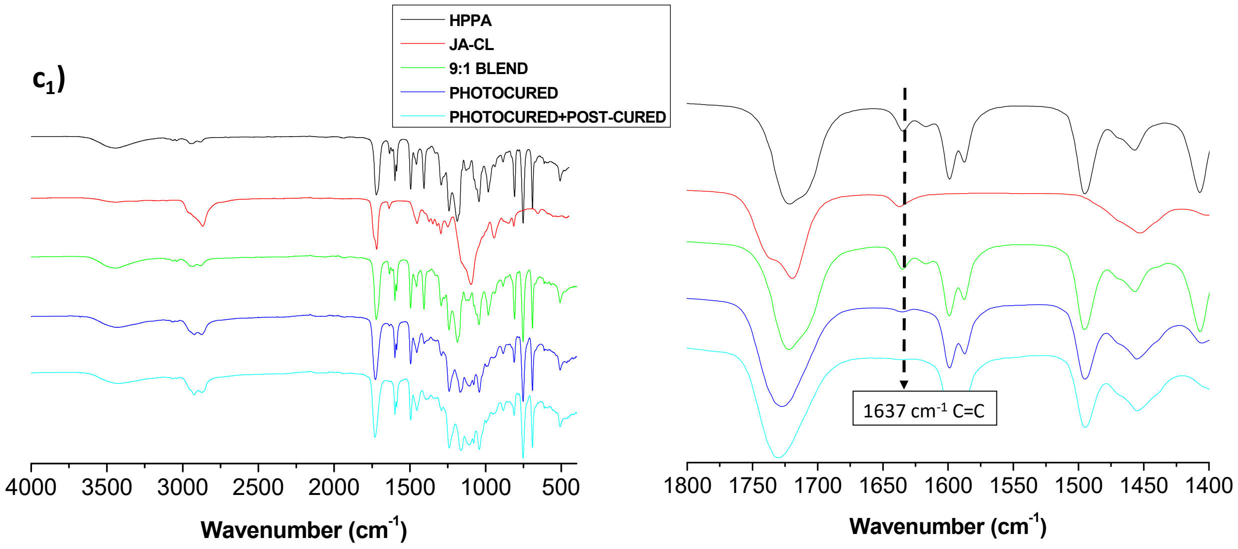
c1)
HPPA
JA-CL
9:1 BLEND
PHOTOCURED
PHOTOCURED+POST-CURED
4000 3500 3000 2500 2000 1500 1000 500
Wavenumber (cm-1)
1637 cm-1 C=C
1800 1750 1700 1650 1600 1550 1500 1450 1400
Wavenumber (cm-1)

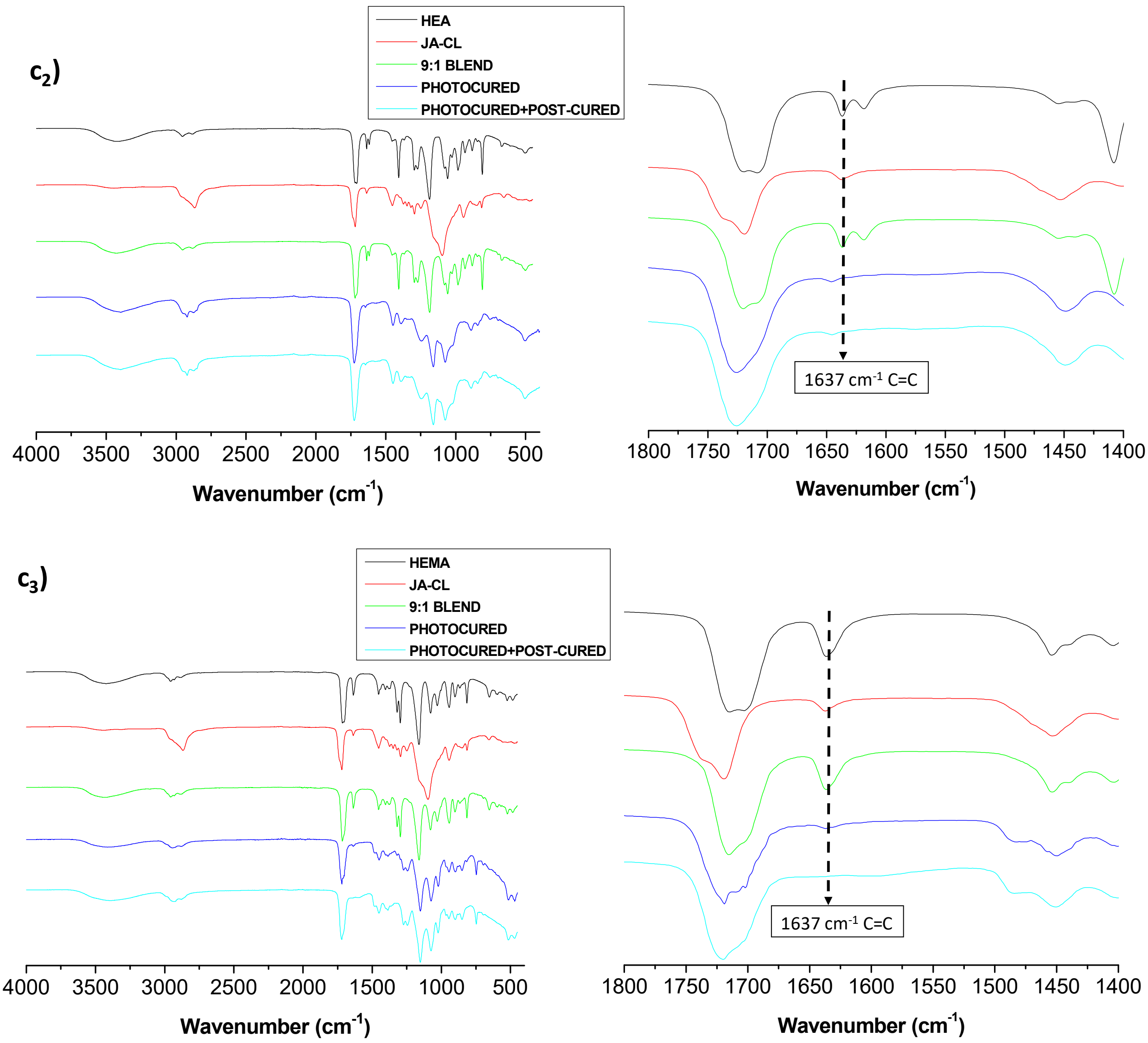

Figure S10. FT-IR spectra of illustrative series of vitrimers to address the success of the photopolymerization and the post-curing steps. On the left, the full spectra are shown, and for clarity purposes, a zoom of the spectra in the region between 1800 cm^{-1} and 1400 cm^{-1} is depicted on the right. In each graph it is shown the spectra of the monomer (black line), the crosslinker (red line), the blend (green line), the vitrimer after photopolymerization (dark blue line) and after post-curing (light blue line): a_1) VitHEMA$_9$:JA-CL$_1$ a_2) VitHEMA$_9$:TTDA-CL$_1$ a_3) VitHEMA$_9$:PA-CL$_1$; b_1) VitHEMA$_2$:PA-CL$_1$ b_2) VitHEMA$_5$:PA-CL$_1$ b_3) VitHEMA$_9$:PA-CL$_1$; c_1) VitHPPA$_9$:JA-CL$_1$ c_2) VitHEA$_9$:JA-CL$_1$ c_3) VitHEMA$_9$:JA-CL$_1$, being a) Comparison between different crosslinkers, b) Comparison when changing the monomer:crosslinker proportion and c) Comparison between different monomers.

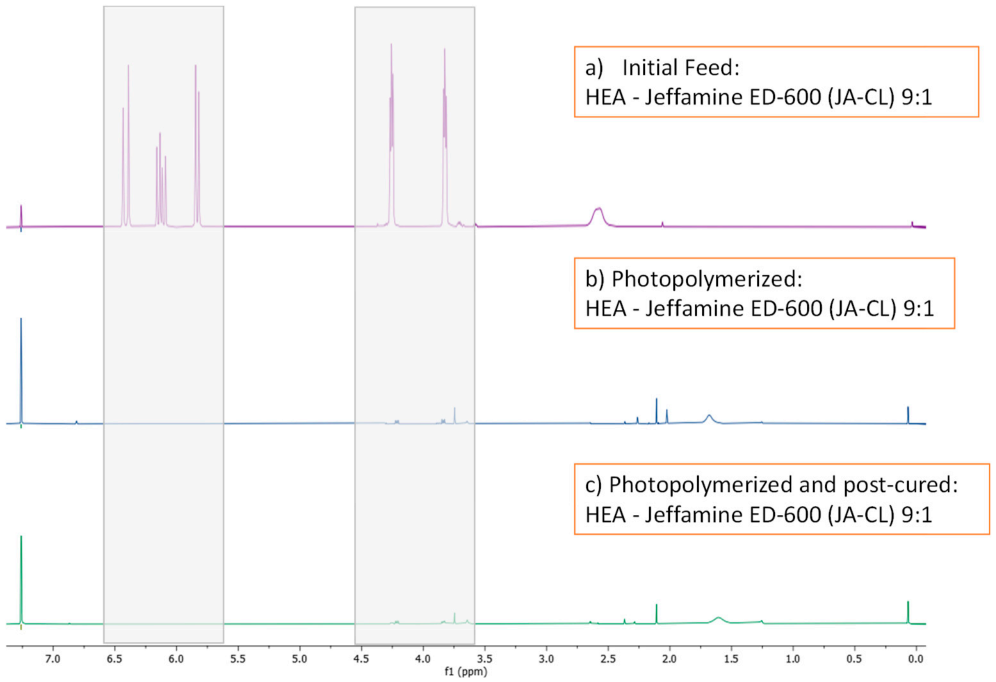

Figure S11. 1H NMR of: a) Initial feed employed comprising HEA and the crosslinker based on Jeffamine in a 9:1 ratio. b) Extract obtained from the photopolymerized sample using chloroform during 48h. c) Extract obtained from a sample that has been both photopolymerized and post-cured using chloroform during 48h.

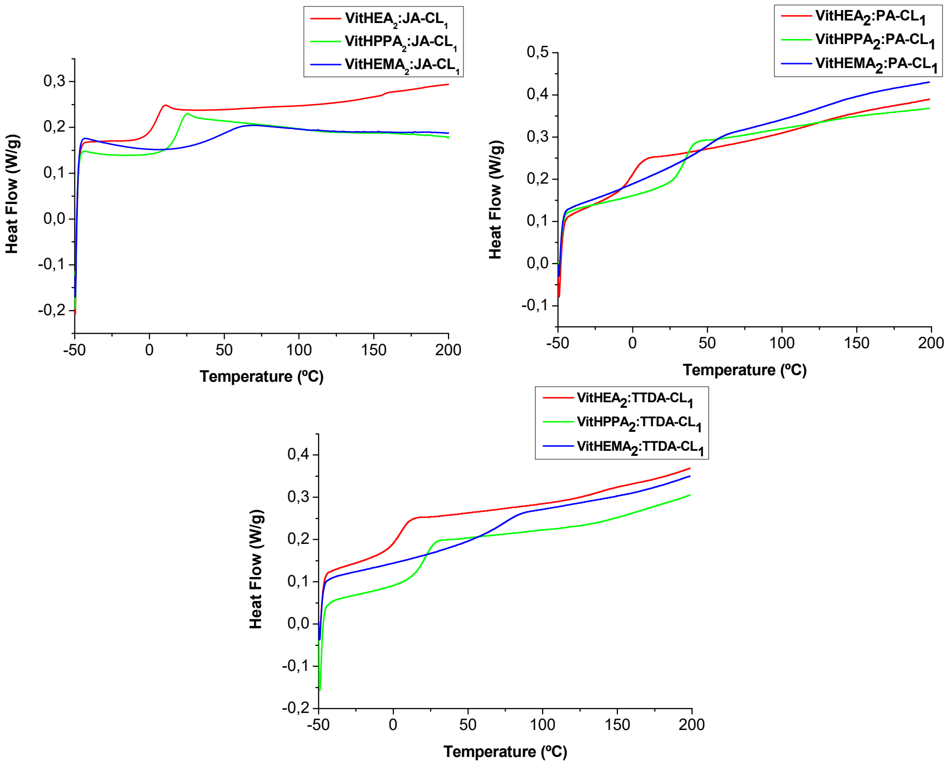

Figure S12. Illustrative DSC curves comparing the effect on the glass transition temperature when the monomer varies.

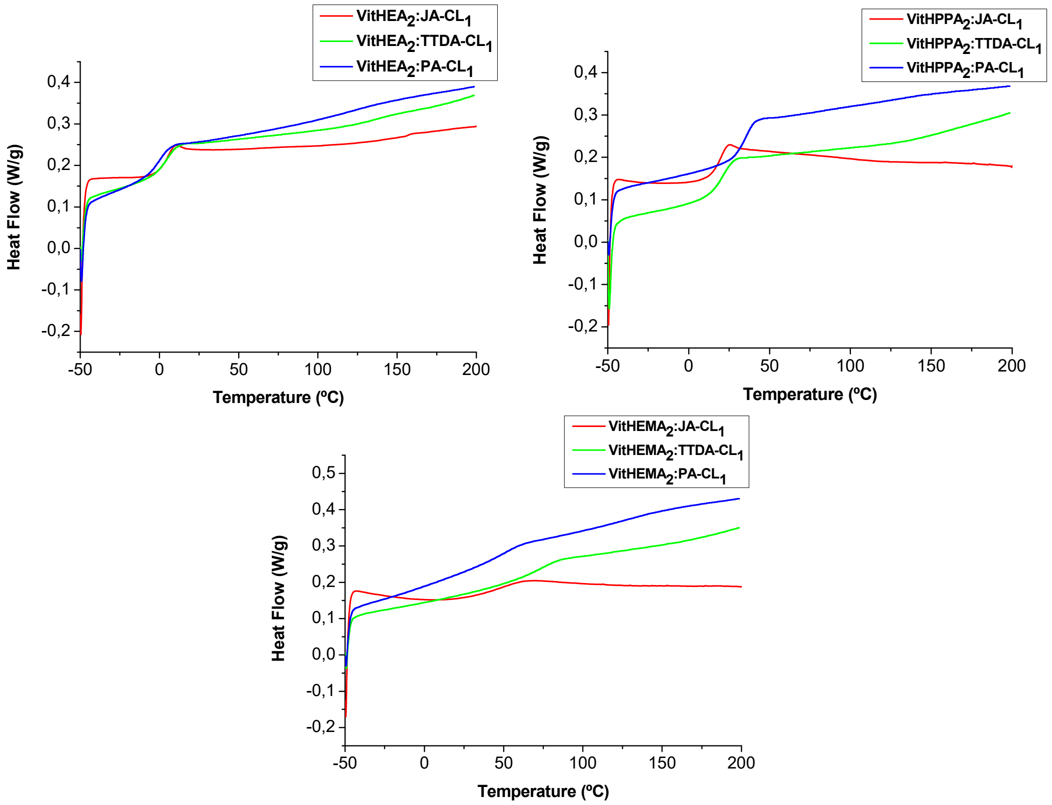

Figure S13. Illustrative DSC curves comparing the effect on the glass transition temperature while the crosslinker varies.

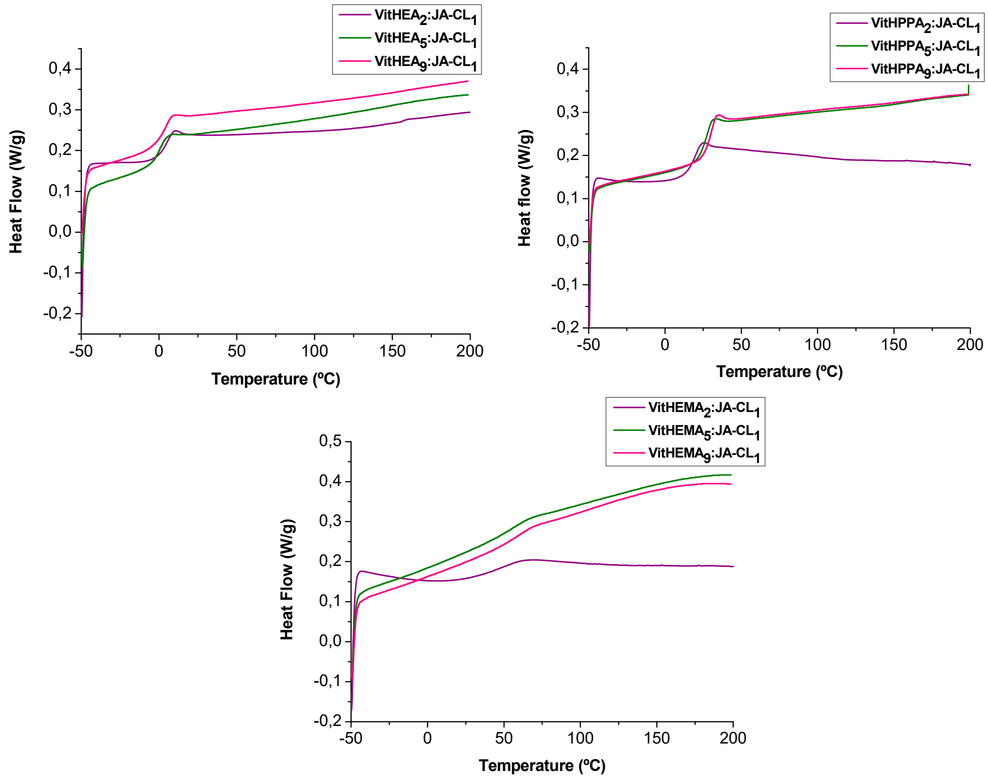

Figure S14. Illustrative DSC curves comparing the effect of varying the monomer:crosslinker proportion on the glass transition temperature.

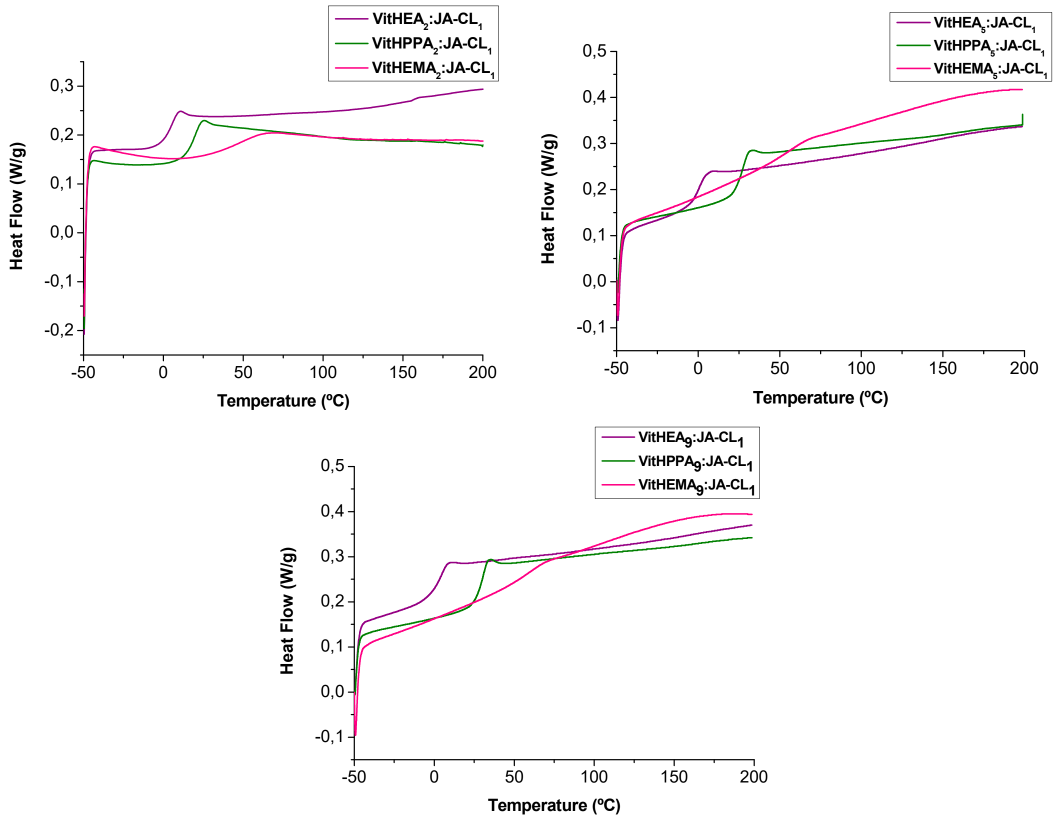

Figure S15. Illustrative DSC curves comparing the effect on the glass transition temperature when the monomer varies.

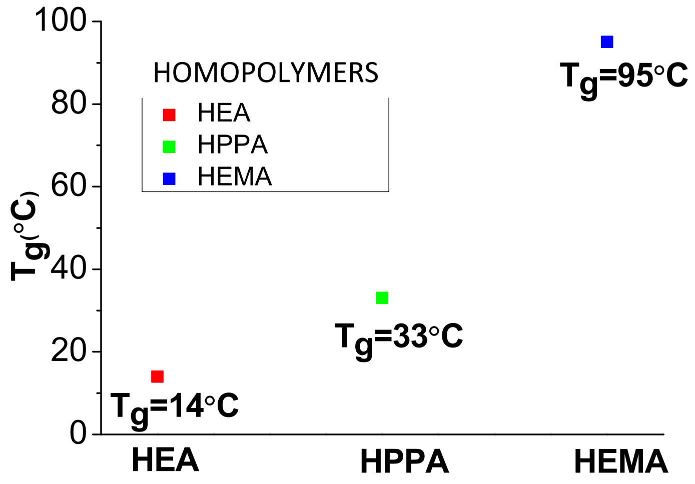

Figure S16. Glass transition temperature (Tg) values obtained from the DSC curves of the homopolymers formed by each monomer: HEA (red), HEMA (green), and HPPA (blue).

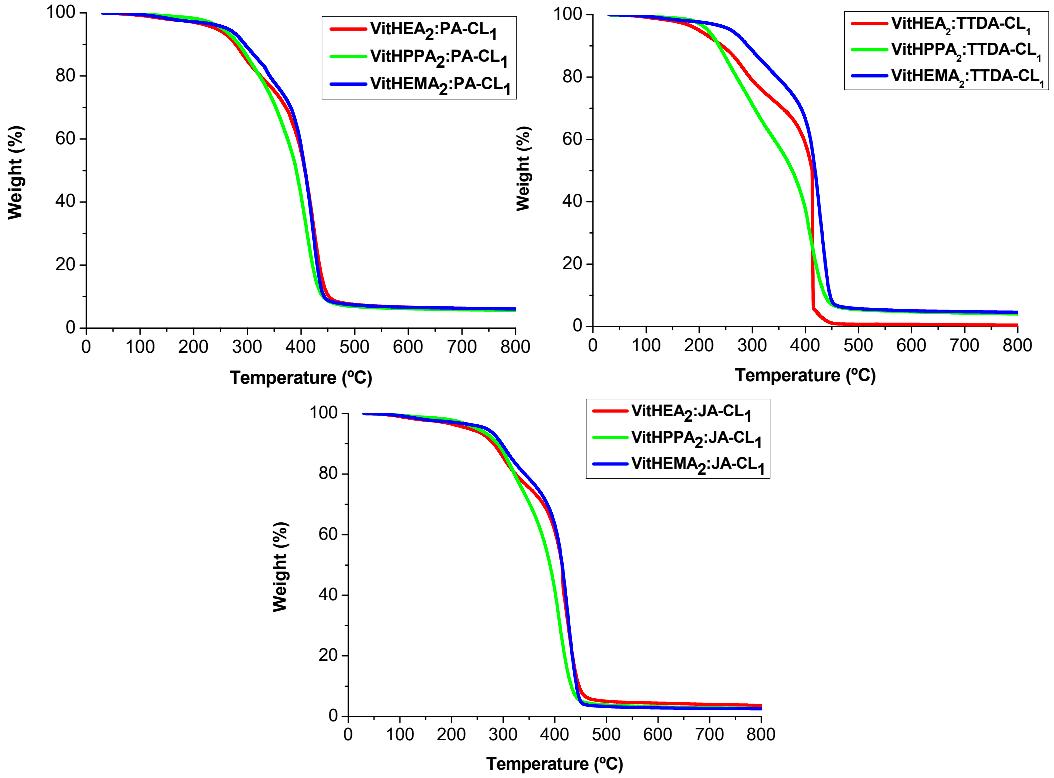

Figure S17. Illustrative thermograms comparing the effect of varying the monomer with different crosslinkers.
